# Biomimetic retractable DNA nanocarrier with sensitive responsivity for efficient drug delivery and enhanced photothermal therapy

**DOI:** 10.1186/s12951-023-01806-5

**Published:** 2023-02-09

**Authors:** Yuanhuan Yang, Xueting Cai, Menglin Shi, Xiaobo Zhang, Yang Pan, Yue Zhang, Huangxian Ju, Peng Cao

**Affiliations:** 1 School of Pharmacy, Nanjing University of Chinese Medicine, Nanjing, 210023 China; 2 Affiliated Hospital of Integrated Traditional Chinese and Western Medicine, Nanjing University of Chinese Medicine, Nanjing, 210028 China; 3 State Key Laboratory of Analytical Chemistry for Life Science, School of Chemistry and Chemical Engineering, Nanjing University, Nanjing, 210023 China; 4 Zhenjiang Hospital of Chinese Traditional and Western Medicine, Zhenjiang, 212002 China

**Keywords:** DNA nanotechnology, Drug delivery, Photo responsive, Biomimetic nanomaterials, Cancer therapy

## Abstract

**Background:**

The coalition of DNA nanotechnology with diversiform inorganic nanoparticles offers powerful tools for the design and construction of stimuli-responsive drug delivery systems with spatiotemporal controllability, but it remains challenging to achieve high-density oligonucleotides modification close to inorganic nanocores for their sensitive responsivity to optical or thermal signals.

**Results:**

Inspired by *Actinia* with retractable tentacles, here we design an artificial nano-*Actinia* consisted of collapsible DNA architectures attached on gold nanoparticle (AuNP) for efficient drug delivery and enhanced photothermal therapy. The collapsible spheroidal architectures are formed by the hybridization of long DNA strand produced in situ through rolling circle amplification with bundling DNA strands, and contain numerous double-helical segments for the intercalative binding of quercetin as the anti-cancer drug. Under 800-nm light irradiation, the photothermal conversion of AuNPs induces intensive localized heating, which unwinds the double helixes and leads to the disassembly of DNA nanospheres on the surface of AuNPs. The consequently released quercetin can inhibit the expression of heat shock protein 27 and decrease the thermal resistance of tumor cells, thus enhancing photothermal therapy efficacy.

**Conclusions:**

By combining the deformable DNA nanostructures with gold nanocores, this *Actinia*-mimetic nanocarrier presents a promising tool for the development of DNA-AuNPs complex and opens a new horizon for the stimuli-responsive drug delivery.

**Graphical Abstract:**

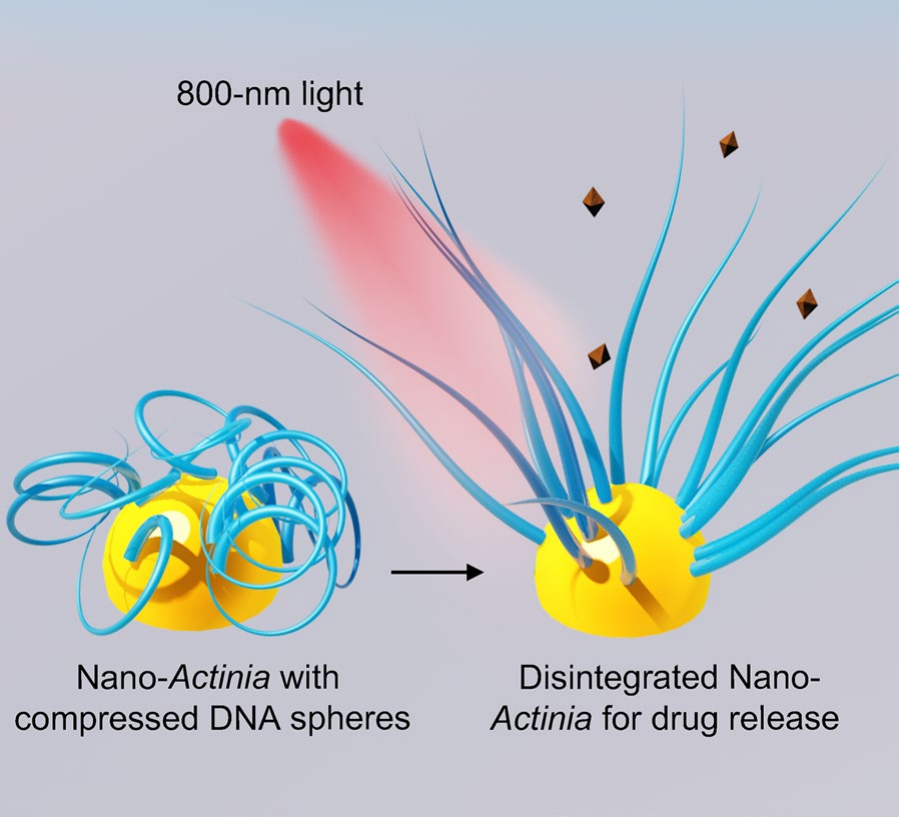

**Supplementary Information:**

The online version contains supplementary material available at 10.1186/s12951-023-01806-5.

## Background

Stimuli-responsive drug delivery systems have attracted substantial attention over the past decades considering their ability to secure the drug accumulation in specific cells and eliminate off-target toxicity [1–3]. As an emerging nanotechnology, DNA have become one of the most prevalent elements for the design and construction of drug delivery systems owing to the programmable intermolecular interactions of Watson–Crick base pairing [4], accurate molecular recognition [5], automated chemistry synthesis and convenient functional group modification [6, 7]. Multifarious 2D or 3D DNA architectures such as nanotrains [8] and nanococoons [9] have been developed to achieve cellular targeting or the controlled cargo release.

Owing to the variable modifications and unique properties including optical [10], thermal [11] or catalytic [12] behaviors, inorganic materials such as gold nanoparticles (AuNPs) or conversion nanoparticles gather much talent in drug delivery and cancer therapy [13, 14], and the invention of DNA nanostructures with inorganic nanocores offers new prospects for the biomedical applications of nucleic acid architectures [15]. These unique coalitions possess both programmability of oligonucleotides and physical properties of inorganic materials, which endow nanocarriers with smart characteristics and external manipulability like near-infrared (NIR) photo responsiveness [16] or magnetic targeting [17].

**Scheme 1:**
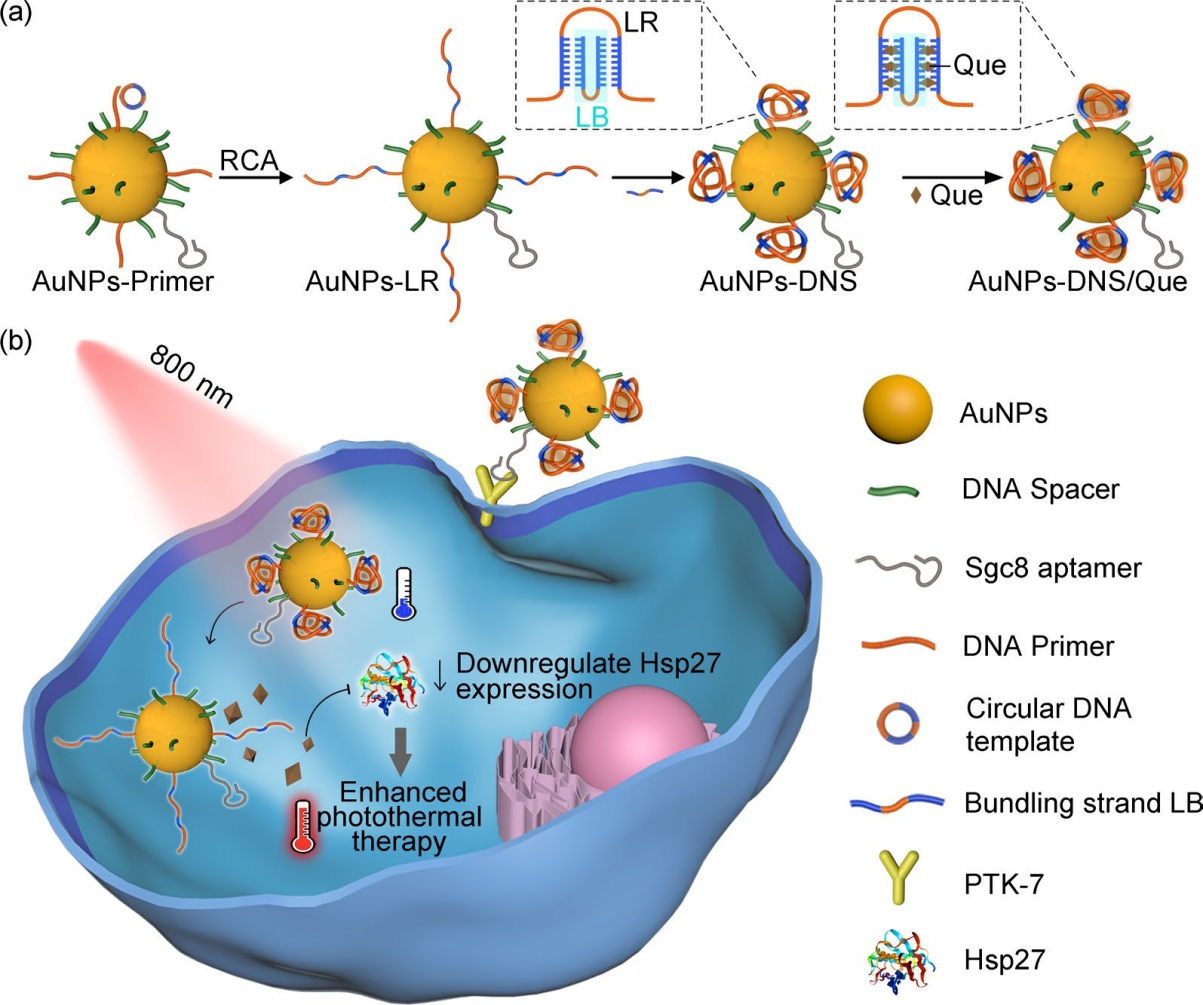
**a** Schematic illustration of AuNPs-DNS/Que synthesis. **b** Aptamer-mediated tumor cell targeting as well as 800-nm light responsive drug release and enhanced photothermal therapy

To acquire an ideal stimulus-responsive delivery system, adequate loading amount of drug molecules [18] and high release efficiency [19, 20] are the most indispensable prerequisites, which demand multiple oligonucleotides for the package of cargos and close distance between DNA and inorganic nanocores for efficient signal transmission. Although DNA origami has been used in some cases to obtain high loading capacity [21, 22], the extended DNA nanostructures made it difficult to respond to the optical or thermal signal generated from inorganic nanocores for controllable drug release. Inspired by the retractable tentacles of *Actinia*, we designed an artificial nano-*Actinia* constituted from* in situ* formation of deformable DNA nanostructures on a gold nanoparticle (AuNP) for efficient drug loading and photo-controlled release. AuNPs acted as the core for absorbing 800-nm light and converting it to localized heat due to the inherent photothermal effect [23], and the elongated tentacles were constructed using long DNA strands (LR), which were produced *in situ* on AuNP by rolling circle amplification (RCA) and could hybridize with bundling DNA strands (LB) to form a shrunk spheroidal architecture (Scheme 1a). The compressed DNA nanosphere (DNS) incorporated with plenty double helixes enabled the efficient loading of anti-cancer drug quercetin (Que) through intercalative binding [24, 25], which contributes to the liberation of quercetin in the condition of helix unwinding. Under 800-nm light irradiation, the photothermic effect of AuNP induced the localized heating and thus facilitated despiralization of the helixes to release quercetin (Scheme 1b). The shrinkage deformation strategy not only guaranteed the enormous quantity of double helixes for Que loading, but also brought the DNA structures close to the nanocore for getting high sensibility of thermal response, which could inordinately enhance the efficiency of drug release under NIR light irradiation. The released quercetin could down-regulate the expression level of heat shock protein 27 (Hsp27) [26] to enhance the efficacy of photothermal therapy (PTT) through decreasing the thermal resistance of tumor cells. Benefiting from the *Actinia*-mimetic design, this AuNPs-DNS/Que could help to resolve the conflict between modification of multiple oligonucleotides and sensitive interactive responsivity to signals of nanocores, which opens up new paths and opportunities for the applications of DNA nanostructures in controllable drug delivery and anti-cancer therapeutics.

## Methods

### Materials and reagents

T4 DNA ligase, 10 × T4 DNA buffer and bovine serum albumin were purchased from Beyotime Biotechnology (Shanghai, China). Exonuclease I, exonuclease III, phi29 DNA polymerase and dNTPs were purchased from Novoprotein Scientific Inc. (Suzhou, China). HAuCl_4_, sodium citrate, NaCl, sodium dodecyl sulfate (SDS), (2-carboxyethyl) phosphine hydrochloride (TCEP), uranyl acetate and quercetin were purchased from Aladin Ltd (Shanghai, China). PBS, MTT cell proliferation and cytotoxicity detection kit, annexin V-FITC/PI apoptosis detection kit and hematoxylin–eosin (HE) dye solution were purchased from Keygen Biotech (Nanjing, China). Hsp27 enzyme-linked immunosorbent assay (ELISA) kit was purchased from Jin Yibai Biological Technology (Nanjing, China). DNA ladder marker was purchased from Takara Biomedical Technology (Beijing) Co., Ltd. (Beijing, China). All DNA strands were synthesized and purified by Sangon Biotech Co., Ltd (Shanghai, China), and their specific sequences and label information were listed in Additional file 1: Table S1.

### Apparatus

Zeta potential analysis and dynamic light scattering (DLS) analysis were conducted on ZetaPlus 90 Plus/BI-MAS (Brook haven, USA). Transmission electron microscopic (TEM) images were captured on JEM-2800 transmission electron microscope (JEOL Ltd., Japan). MTT and ELISA assays were conducted on Victor X3 Multimode Plate Reader (PerkinElmer, USA). The gel electrophoresis was performed with Mini-Protean Tetra Cell and PowerPac™ Basic Power Supply (Bio-Rad, USA). The intracellular fluorescence images were acquired through TCS SP5 confocal laser scanning microscope (CLSM) (Leica, Germany). Flow cytometry was accomplished on Coulter FC-500 flow cytometer (Beckman-Coulter, USA). The UV–Vis absorption spectra were recorded with UV-3600N UV/VIS spectrophotometer (Shimadzu, Japan). The fluorescence spectra were recorded with F-4700 spectrofluorophotometer (Hitachi, Japan).

### Synthesis of DNA nanosphere (DNS)

The circular DNA template was first prepared according to a previously reported method [27]. 4.2 μL 100 μM phosphorylated linear DNA and 4.2 μL 100 μM ligation DNA were mixed to react at 95 °C for 4 min. After slowly cooling down to room temperature over 4 h, the mixture was added with 1 μL 400 U μL^−1^ T4 DNA ligase, 2 μL 10 × T4 DNA buffer and 8.6 μL DI water, and incubated at 25 °C for 16 h. Then the T4 DNA ligase was denatured by heating at 65 °C for 10 min, and 4 μL exonuclease I (20 U μL^−1^) and 4 μL of exonuclease III (100 U μL^−1^) was added to react for 8 h at 37 °C for the decomposition of ligation DNA. Afterwards, the product was heated and kept at 80 °C for 15 min to denature the DNA exonucleases, and the obtained circular DNA template was stored at 4 °C for the further use.

The long-chain DNA LR was synthesized through RCA reaction. Typically, 10 μL circular DNA template (3 μM) was mixed with 0.5 μL 100 μM DNA primer and then annealed at 95 °C for 5 min, followed by cooling to room temperature. Afterwards, the mixture was added with 0.2 U μL^−1^ phi29 DNA polymerase, 0.4 μg μL^−1^ bovine serum albumin and 0.1 mM dNTPs and incubated at 37 °C for 5 h in 150 μL phi29 reaction buffer. After reaction, the product was heated and kept at 65 °C for 10 min to inactivate the phi29 DNA polymerase, and purified through ultrafiltration using 100kD cutoff membrane. Finally, 10 μL bundling strand LB (100 μM) was added and incubated at 37 °C for 2 h to hybridize with LB and form the spherical DNS.

### Preparation of AuNPs

AuNPs were synthesized referring to the classical sodium citrate reduction method [28]. Briefly, 50 mL HAuCl_4_ (0.01%) was heated to boiling and added with 0.5 mL sodium citrate (1%). Then the solution was refluxed for 10 min and cooled down to room temperature.

### Modification of DNA primer

Thiol-modified primer DNA was conjugated on the AuNPs according to the previous reported procedure [29]. First of all, 10 μM thiolated primer was activated through incubation with 10 mM TCEP for 2 h at room temperature, and thiol group anchored DNA spacer and Sgc8 aptamer were also pretreated with the same procedure. Then 1 mL AuNPs (1 nM) were added with the mixture of 10 μL primer DNA (10 μM), 10 μL DNA spacer (50 μM) and 10 μL Sgc8 aptamer (10 μM) stirred for 20 min, followed by adding with 10 μL SDS (10%), 100 uL 0.1 M PBS (pH = 7.4) and 50 μL 2.0 M NaCl. The resulting mixture was stirred for 30 min at room temperature and the addition of 2.0 M NaCl (60 μL) was repeated twice with 30-min interval. Then the final mixture was stirred for 24 h at room temperature and the obtained primer/spacer/aptamer modified AuNPs were collected through centrifugation.

To detect the modification amounts of DNA primers, DNA spacers and Sgc8 aptamers on AuNPs, FAM-labeled primer (Primer_FAM_), Cy3-labeled spacer (Spacer_Cy3_) and Cy5-labeled aptamer (Aptamer_Cy5_) was used as substitutions respectively, and the synthesized fluorescent nanoparticles were centrifuged (8000 rpm, 20 min) to collect the supernatants for fluorescence detecion. The reaction quantities were calculated through the comparison with corresponding calibration curves.

### Preparation of AuNPs-DNS

The pre-synthesized circular DNA template (50 nM) was added with primer modified AuNPs (1 nM) and annealed at 90 ºC for 5 min, followed by cooling down to room temperature slowly. The obtained template conjugated AuNPs were centrifuged (8000 rpm, 20 min) and then mixed with phi29 DNA polymerase (0.2 U mL^−1^), bovine serum albumin (0.4 mg mL^−1^), dNTPs (0.1 mM) and 10 × phi29 reaction buffer and reacted for 3 h. After that, the mixture was incubated at 65 ºC for 10 min, centrifuged (8000 rpm, 20 min) and washed with PBS buffer for 3 times to get long chain LR attached AuNPs (AuNPs-LR). Finally, 2 μM bundling strand LB was mixed with 1 nM AuNPs-LR and reacted at 37 ºC for 2 h with a slow shaking at 100 rpm. The obtained AuNPs-DNS was centrifuged (8000 rpm, 20 min) and washed with PBS buffer for 3 times.

To optimize the modification amounts of DNA primer, DNA spacer and Sgc8 aptamer, different concentrations of these DNA strands were added into 1 nM AuNPs solutions to synthesize AuNPs-DNS according to the same procedure above.

To verify the formation of DNS on AuNPs, bundling strand labeled with Cy3 and BHQ at 3' and 5' respectively (LB_Cy3/BHQ_) was used as substitution to synthesize AuNPs-DNS_Cy3/BHQ_ according to the same approach above. Then the Cy3 fluorescence of the mixture was detected and compared with the initial LB_Cy3/BHQ_ solution with a concentration of 2 μM.

The Cy5 and Cy3/BHQ labeled AuNPs-DNS were synthesized with the same approach except that Cy5 labeled Sgc8 aptamer (Aptamer_Cy5_) and Cy3/BHQ labeled bundling strand LB_Cy3/BHQ_ were used as substitutions respectively.

### Quercetin loading

50 μL 20 μM quercetin DMSO solution was added into 1 mL 0.1 nM AuNPs-DNS and incubated at room temperature for 6 h with slight shaking and then centrifuged at 8000 rpm for 20 min. The fluorescence intensity of quercetin at 503 nm in the supernatant after reaction was detected under 250-nm excitation, and the loading amount was calculated in comparison with the calibration curve of quercetin.

### Transmission electron microscopic (TEM) imaging of DNS

10 μL of the prepared DNS, AuNPs-DNS or AuNPs-DNS/Que was dropped on a carbon supported membrane (ZhongJing KeYi Technology Co., Ltd., China) and stained with 2% uranyl acetate for 10 min. After washing with DI water for 3 times, the carbon supported membrane was dried under nitrogen for TEM imaging.

### Quercetin release

To prove the NIR light-controlled drug release, 1 mL 0.1 nM AuNPs-DNS/Que was irradiated with 800-nm laser (2 W cm^−2^) for different times. Afterwards, the nanoparticles were centrifuged to detect the fluorescence of quercetin in the supernatants. The release rate was calculated through comparing the fluorescence intensities of supernatants with the calibration curve of quercetin.

### Stability examination

0.1 nM AuNPs-DNS was immersed in PBS or PBS buffer containing 10% FBS and incubated for 24 h at 37 °C to detect the hydration size through DLS analysis. Besides, 0.1 nM AuNPs-DNS/Que was also incubated in PBS or FBS buffer to monitor the leakage of quercetin through measuring the fluorescence intensities of supernatants and comparing with the calibration curve.

### Cell culture

MCF-7 cells and MCF-10a cells (Keygen Biotech, China) were cultured in RPMI 1640 medium complemented with 10% FBS and 1% Penicillin–Streptomycin Solution (100 μg mL^‒1^) at 37 °C in a humidified atmosphere containing 5% CO_2_. The cell numbers were counted through Countess^®^ II automated cell counter (Invitrogen, USA).

### Tumor cell targeting

1 × 10^4^ MCF-7 cells or MCF-10a cells were seeded into a glass dish and treated with 1 nM Cy5 labeled AuNPs-DNS prepared by Aptamer_Cy5_ at 37 °C for 6 h. After washing with PBS for 3 times, the treated cells were collected to detect the fluorescence signal of AuNPs-DNS_Cy5_ through flow cytometer over FL3 channel. Moreover, MCF-7 cells treated with 1 nM control AuNPs-DNS which was synthesized using Cy5 labeled random sequence (Control_Cy5_) instead of the Aptamer_Cy5_ were also imaged as control.

### Investigation of endocytosis pathway

Different inhibitors of cell endocytosis, sucrose (450 mM), methyl-β-cyclodextrin (50 µM), genistein (200 µg mL^‒1^), wortmannin (50 nM) and NaN_3_ (10 mM) were added into MCF-7 cells and incubated for 30 min to block corresponding internalization processes including clathrin, lipid raft, caveolae, macropinocytosis and energy dependent endocytosis respectively. Then the cells were incubated with 1 nM AuNPs-DNS_Cy5_ for 6 h and washed with PBS for three times. The fluorescence intensities of MCF-7 cells were measured through the flow cytometric analyses over FL3 to calculate the AuNPs-DNS uptake rates.

### CLSM imaging

To validate the intracellular NIR light responsivity of AuNPs-DNS, 1 × 10^4^ MCF-7 cells were seeded in a glass bottom dish for 24 h at 37 °C and then incubated with 1 nM biocolor labeled AuNPs-DNS synthesized from Aptamer_Cy5_ and LB_Cy3/BHQ_ for 6 h at 37 °C. After washing with PBS for three times, the treated cells were irradiated with 800-nm light for 10 min, and the fluorescence of Cy5 and Cy3 was observed from 650 to 700 nm under 633 nm-excitation and from 560 to 620 nm with the excitation at 543 nm respectively.

### Cytotoxicity assay

MCF-7 cells were cultured in a 96-well plate (Corning Inc. USA) with a density of 1 × 10^4^ cells per chamber for 24 h, and then 1 nM AuNPs-DNS or AuNPs-DNS/Que was added into each well to incubate at 37 °C for 6 h. Finally, the treated cells were washed with PBS for three times to remove redundant nanoparticles, and the cell viability was analyzed through MTT assay according to the instruction procedure. The cell viability of MCF-7 cells treated with 10-min 800-nm light exposure were also detected with the same procedure.

### Intracellular therapeutic efficiency

After MCF-7 cells were seeded into the 96-well plate (Corning Inc. USA) with a culture density of 1 × 10^4^ cells per chamber for 24 h, 1 nM AuNPs-DNS/Que was added and incubated for 6 h. Then the MCF-7 cells were washed with PBS for three times and exposed under 800-nm light (2 W cm^‒2^) for different times. Finally fresh medium containing 10% FBS was added and the cells were cultured for another 24 h to detect the cell proliferation rate through the MTT cell proliferation and cytotoxicity detection kit according to the instruction procedure.

To further demonstrated the efficient and specific killing effect on tumor cells and good biocompatibility of AuNPs-DNS/Que, different concentrations (0.5 nM, 1 nM, 1.5 nM) of AuNPs-DNS/Que were incubated with MCF-7 cells or MCF-10a cells for 6 h, followed by 10 min irradiation with 800-nm light (2 W cm^‒2^). The cell proliferation rates were evaluated according to the same procedure above.

To verify the better therapeutic efficacy of AuNPs-DNS/Que, different concentrations of quercetin or AuNPs-DNS/Que with the same amounts of quercetin were added and incubated with MCF-7 cells for 6 h. After washing with PBS and irradiation with 800-nm light (2 W cm^‒2^) for 10 min, the treated cells were cultured for another 24 h to conduct the MTT cell proliferation assay. Cells treated with AuNPs-DNS at the same concentrations with AuNPs-DNS/Que were also detected according to the same procedure.

For the apoptosis assay, the MCF-7 cells were treated with 8 μM quercetin, 1 nM AuNPs-DNS or AuNPs-DNS/Que for 6 h and then exposed under 10-min NIR light (2 W cm^‒2^) or not. After incubation in fresh culture medium containing 10% FBS for another 24 h, the treated cells were stained with Annexin V-FITC/PI apoptosis detection kit according to the instruction procedure and analyzed with flow cytometer over FL1 and FL3 channel.

### Evaluation of Hsp27 expression level

MCF-7 cells were seeded in a 24-well plate (Corning Inc, USA) with a density of 5 × 10^5^ cells per well and cultured at 37 °C for 24 h. Then 1 nM AuNPs-DNS or quercetin loaded AuNPs-DNS (AuNPs-DNS/Que) was added and incubated for 6 h. After irradiation with 800-nm light (2 W cm^‒2^), the cells were incubated with fresh medium containing 10% FBS and further cultured for 48 h to detect the cellular expression levels of Hsp27 using enzyme-linked immuno sorbent assay (ELISA) according to the manufacturer's instructions. The cells without any treatment and cells only treated with NIR light were also detected as controls. The expression levels of Hsp27 in MCF-7 cells treated with different concentrations of AuNPs-DNS/Que as well as free quercetin were also evaluated according to the same procedure above.

### In vivo imaging and antitumor efficiency

Pathogen-free female BALB/c nude mice (4–5 weeks old) were purchased from GemPharmatech Co. Ltd. (Nanjing, China). All of the mice had free access to water and rodent chow. All experiments were conducted according to the NIH guidelines for the care and use of laboratory animals (NIH Publication no. 85–23 Rev. 1985) and approved by the Experimental Animal Center of Nanjing University of Chinese Medicine (approval number: 202103A025). To establish a mouse model of MCF-7 tumor xenograft, 1 × 10^6^ MCF-7 cells were subcutaneously inoculated into the underarm position of the female nude mice. The tumor volumes were calculated through the formula V = (L × W^2^)/2, where L and W are the length and width of the tumor respectively.

To evaluate the in vivo photothermal effect of AuNPs-DNS/Que, the tumor-bearing mice were intratumorally injected with 150 μL of 1 nM AuNPs-DNS/Que or saline and then exposed under 800-nm light (1 W cm^−2^) for 10 min to capture the thermographic images through a near infrared thermal imager (FLIR-E64501, FLIR Systems Inc., USA).

When the tumor grew to a volume over 80 mm^3^, the tumor-bearing mice were divided into six groups randomly, and the weights as well as tumor volumes were measured and recorded. Afterwards, different groups of mice were intratumorally injected with 150 μL of (1) (2) saline, (3) (4) 1 nM AuNPs-DNS, (5) (6) 1 nM AuNPs-DNS/Que and (5) (6) 8 μM free quercetin. At 6 h after injection, the tumors on the mice of groups (2), (4), (6) and (8) were exposed under 800-nm light (1 W cm^−2^) for 10 min, while the groups (1), (3), (5) and (7) served as controls and did not experience any NIR irradiation. The injection and irradiation was conducted at day 3 and day 6 repeatedly. During this time, the mice were weighted every 2 days along with the tumor volume monitoring. At Day 14, all the mice were euthanized and representative photos of these tumor-bearing mice were taken. Then the tumor and major organs including heart, spleen, kidney, liver, and lung were collected, washed with saline, and then fixed in the 4% paraformaldehyde solution for the histopathological analysis. After imbedding in paraffin blocks, the paraformaldehyde-fixed organs were sliced to 5 μm sections, and the sections were stained through HE dye solution and visualized with optical microscope (Olympus BX51, Japan).

To evaluate the biodistribution of AuNPs-DNS, the tumor-bearing mice were intravenously injected with 150 μL of saline or 1 nM Cy5 labeled AuNPs-DNS synthesized from Aptamer_Cy5_ (AuNPs-DNS_Cy5_) and imaged at 6 h after injection on the IVIS Lumina XR III in vivo imaging system (PerkinElmer, USA). Then major organs including heart, spleen, kidney, liver, and lung were also collected and imaged according to the same procedure.

## Results and discussion

### Preparation and characterization of nano-Actinia

**Fig. 1:**
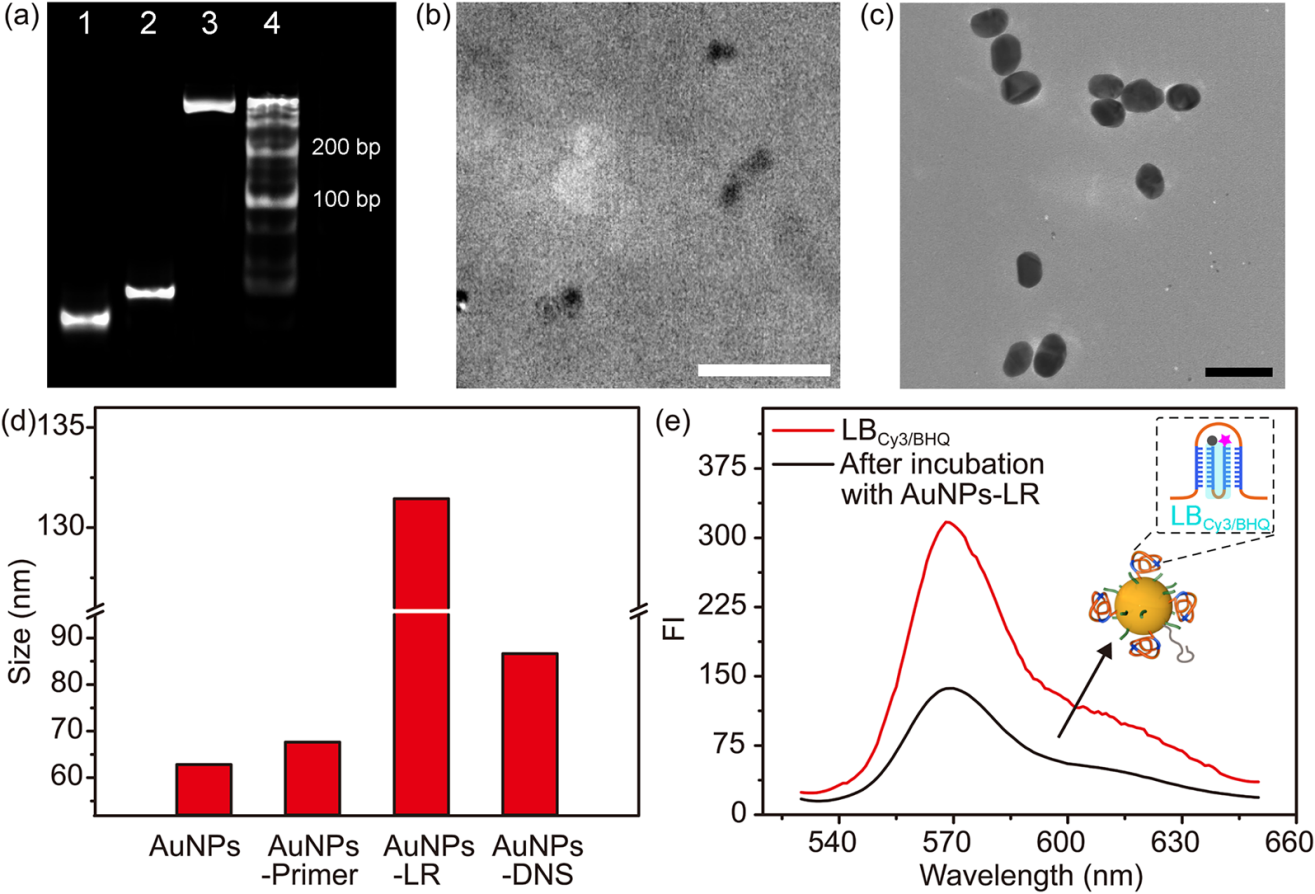
**a** PAGE analysis of DNA primer, circular DNA template, RCA product and DNA ladder marker (lanes 1–4). TEM images of **b** DNS (scale bar: 50 nm) and **c** AuNPs (scale bar: 200 nm). **d** Variation of Hydration diameter during assembly of AuNPs-DNS. **e** Fluorescence spectra of LB_Cy3/BHQ_ before and after incubation with AuNPs-LR

The assembly of DNA nanospheres (DNS) through RCA reaction and then hybridization with the bundling strand (LB) was firstly validated in buffer solution. First of all, the circular DNA template was synthesized using the template sequence (phosphorylated linear DNA), ligation DNA and T4 DNA ligase according to the previous report [30], and then DNA primer hybridized with the prepared circular DNA template to get extended and turn into a long single strand (LR) with plenty of repeating sequences in the presence of DNA polymerase and dNTPs. The polyacrylamide gel electrophoresis (PAGE) analysis indicated the formation of circular DNA template and LR from the observation of the bands with lower mobility (Additional file 1: Figure S1 and Fig. 1a, lane 3). After incubation with LB at 37 ºC for 2 h, the long strand was folded into a compact spheroidal structure with a diameter of 8.3 nm (Fig. 1b) and a hydration diameter of 15.2 nm (Additional file 1: Figure S2).

AuNPs were synthesized using a previously reported procedure [28], which represented a monodispersed particle size of ~ 54 nm (Fig. 1c). The thiol-terminated DNA primers were assembled on the surface of AuNPs via Au-thiol interaction [29], which led to the variation of zeta potential from −5.34 mV to −14.66 mV (Additional file 1: Figure S3a) and the increase of hydration diameter from 62.9 nm to 67.7 nm (Fig. 1d and Additional file 1: Figure S4). Besides, the appearance of the DNA characteristic peak around 260 nm proved the successful conjugation of oligonucleotides on AuNPs (Additional file 1: Figure S3b).

The extension of DNA primer to form long chain LR through RCA reaction on the surface of AuNPs was convinced from the variation of zeta potential from −14.66 mV to −26.81 mV, and the increase of hydration diameter from 67.7 nm to 131.4 nm (Fig. 1d and Additional file 1: Figure S4). After incubation with bundling strands, the hydration size shrank to 86.7 nm (Fig. 1d and Additional file 1: Figure S4d), implying that the loose and scattered long chain LR on AuNPs was folded into a compacted structure. The folding was also validated with the bundling strands labeled with Cy3 and BHQ at the ends respectively (LB_Cy3/BHQ_). After incubating AuNPs-LR with LB_Cy3/BHQ_, Cy3 fluorescence showed conspicuous decrease (Fig. 1e), suggesting the bending of LB during the formation of DNS. The modification amounts of DNA primer, DNA spacer and Sgc8 aptamer were optimized through the shrinkage of hydration sizes, which showed obvious compression for AuNPs added with (1) 100 nM DNA primer, 500 nM DNA spacer and 100 nM Sgc8 aptamer or (2) 50 nM DNA primer, 550 nM DNA spacer and 100 nM Sgc8 aptamer (Additional file 1: Figure S5), indicating the satisfactory assembly of DNA spheres on AuNPs. Considering that the modification of DNA primers with more quantity, which could generate more strands to form DNA spheres, is beneficial for efficient drug loading, the former synthesis condition was adopted for the subsequent experiments.

To avoid the steric hindrance during RCA reaction, a short oligonucleotide with 20 bases as a DNA spacer was assembled on the surface, and Sgc8 aptamer were also modified simultaneously to achieve the tumor cell targeting through recognization with PTK-7 receptor which is highly expressed on the tumor cell membrane [31, 32]. The modification amounts of DNA primer, DNA spacer and Sgc8 aptamer were detected by virtue of FAM-labeled primer, Cy3-labeled spacer and Cy5-labeled aptamer to be 32, 187 and 24 on each AuNP respectively (Additional file 1: Figure S6).

To evaluate the photothermal effect of AuNPs-DNS, the temperature variation of AuNPs-DNS solution with different concentrations was monitored under 800-nm light exposure at a power output of 2 W cm^−2^, which showed a rapid temperature elevation and reached 60.7 ºC after 10-min irradiation for 1 nM AuNPs-DNS, while the PBS buffer only exhibited an inconspicuous increase from 25.6 ºC to 33.7 ºC (Additional file 1: Figure S7).

**Fig. 2:**
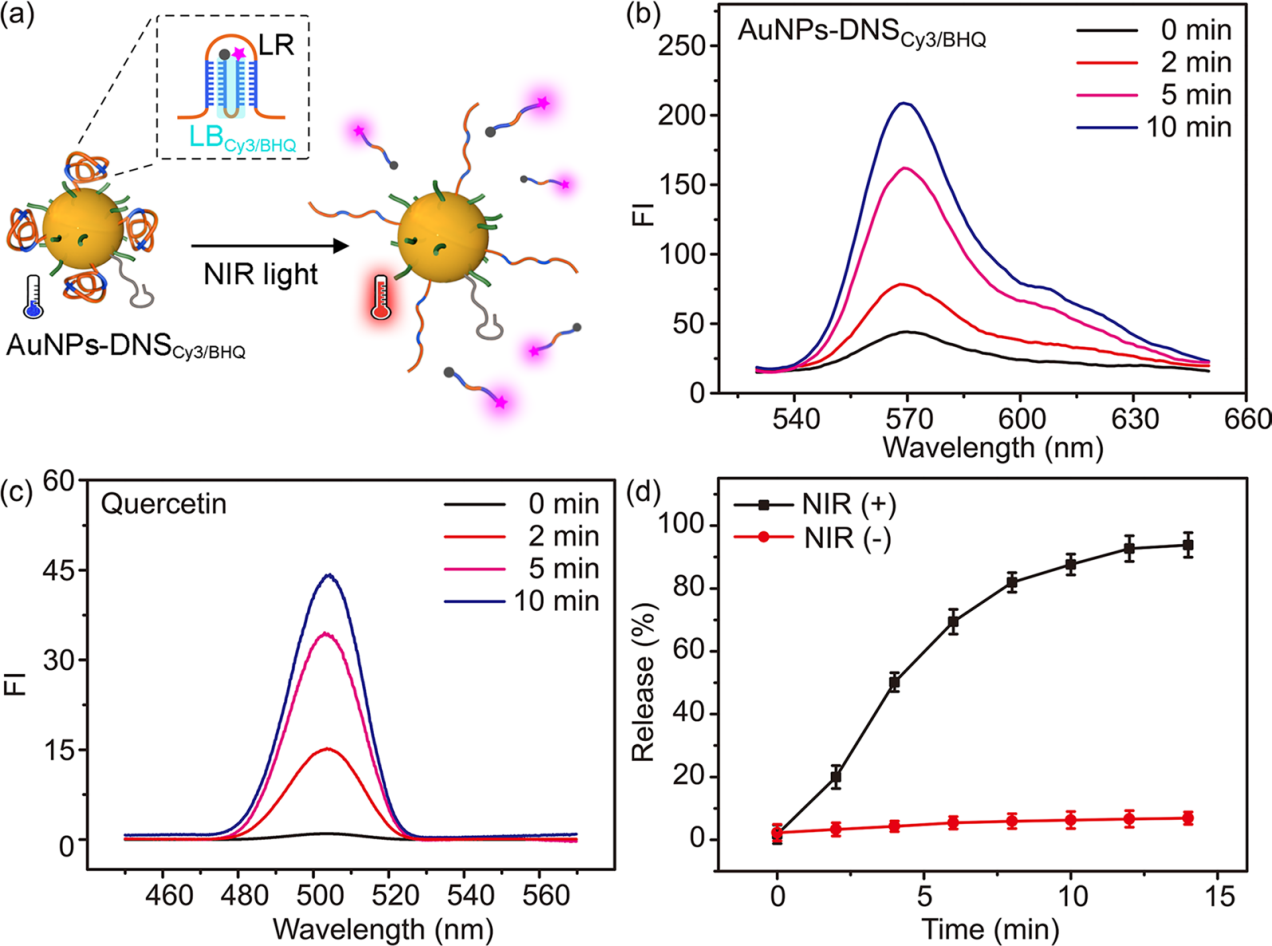
**a** Schematic illustration for the fluorescence recovery of AuNPs-DNS_Cy3/BHQ_ under NIR light irradiation. Fluorescence spectra of **b** AuNPs-DNS_Cy3/BHQ_ and **c** quercetin in the supernatants of AuNPs-DNS/Que upon irradiation for different times. **d** Release percentages of quercetin from AuNPs-DNS/Que under 800-nm light exposure. The data error bars indicate means ± SD (*n* = 3)

The NIR light responsive disassembly of DNS on the AuNPs was verified using AuNPs-DNS_Cy3/BHQ_ through the fluorescence recovery of Cy3, which was initially quenched by the adjacent BHQ after incubation with the bundling strand. Under 800-nm light exposure, the photothermal effect of AuNPs led to localized temperature rise, triggering the dissolution of DNS and liberation of bundling strand, thus the fluorescence of Cy3 gradually recovered (Fig. 2a and b). Additionally, the growing hydration diameter after 10-min exposure was also indicated the 800-nm light responsive disassembly of DNS on AuNPs (Additional file 1: Figure S8).

As a member of natural flavonoids, quercetin exists in multifarious vegetables and fruits, and it has been proved to effectively suppress the expression of heat shock proteins and possess anticarcinogenic properties [26]. Besides, quercetin has a planar structure owing to the intramolecular hydrogen bonds formed from the five hydroxyl groups attached to the carbon atoms [25], which enables it to be conjugated with DNA double helix through the minor groove binding [24, 25], supplying the hybrid-assembled DNS with the ability of quercetin loading. The loading amount of quercetin molecule in each AuNP-DNS/Que was measured to be 8147 through comparing the calibration curve of quercetin with the fluorescence intensity of the supernatant after loading (Additional file 1: Figure S9). The loading of quercetin did not influence the construction of DNS on AuNPs, which could be verified through the TEM images before and after loading (Additional file 1: Figure S10). After the NIR light responsive disassembly of DNS, the loaded quercetin was released from AuNPs-DNS/Que. The supernatants of AuNPs-DNS/Que upon irradiation for different times exhibited an obvious increase of quercetin fluorescence within merely 2 min (Fig. 2c), and the accumulative release percentage of quercetin sustainably elevated as the temperature of AuNPs-DNS/Que solution rose under 800-nm light exposure (Additional file 1: Figure S11), which achieved 87.6% after irradiation for 10 min (Fig. 2d, black line). In contrast, little leakage of quercetin from AuNPs-DNS/Que was observed in the absence of 800-nm light (Fig. 2d, red line), indicating the efficiently NIR light-responsive control for the drug release of the designed *Actinia*-like DNA nanocarrier. To verify the superior responsivity of this collapsible DNA nanosphere, single strands (control ssDNA) with complementary sequences with LR were added instead of LB to generate DNA double helixes on AuNPs as control (Additional file 1: Figure S12a), which displayed an extended hydration diameter of 141.9 nm (Additional file 1: Figure S12b) because of the formation of these rigid and straight DNA segments. Under the irradiation of 800-nm light, a small quantity of Que was released and the release percentage was only 38.6% after 10-min exposure (Additional file 1: Figure S12c). These results provided convincing evidence that the designed compressed DNA architecture close to the AuNPs possessed more sensitive response to the local high temperature and thus facilated the controllable release of loaded drugs.

The stability of the artificial nano-*Actinia* was investigated by incubating it in PBS or 10% fetal bovine serum (FBS) buffer for 24 h, which led to little variation of its hydration diameter (Additional file 1: Figure S13a), indicating the construction integrity of the compressed DNA nanospheres on AuNPs. Additionally, the leakage of quercetin form AuNPs-DNS/Que in PBS or FBS buffer in the absence of NIR light irradiation was also at a very low level (Additional file 1: Figure S13b), declaring the satisfactory stability of AuNPs-DNS/Que during the delivery process.

### Tumor targeting and internalization pathway of nano-Actinia

Breast cancer is one of the well-known malignant tumors and has been identified as an increasing problem in recent years [33]. Here we chose MCF-7 cells, one of the mostly used breast cancer cell lines, to investigate the intracellular properties of AuNPs-DNS. The specific tumor cellular internalization was achieved through the recognition of Sgc8 aptamer assembled on AuNPs to PTK-7 receptor on cell surface. To study the targeting ability of AuNPs-DNS, the aptamer was labeled with Cy5 at 3'-end (Aptamer_Cy5_). It protruded from the AuNPs through 20 T bases at thiol-terminated 5'-end (Table S1), excluding the quenching effect of AuNPs on Cy5 fluorescence. After incubation with Cy5 labeled AuNPs-DNS for 6 h, MCF-7 cells with overexpressed PTK-7 receptor [31, 32] showed obvious fluorescence of Cy5 (Additional file 1: Figure S14a), while PTK-7-negative MCF-10a cells produced negligible fluorescence of Cy5. To further illustrate the targeting ability of Sgc8 aptamer, control AuNPs-DNS (AuNPs-DNS_Control_) were synthesized using Cy5 labeled DNA with random sequence (Control_Cy5_) as the substitution for aptamer-Cy5. The treatment of MCF-7 cells with AuNPs-DNS_Control_ showed negligible fluorescence of Cy5 as those for control and PTK-7-negative MCF-10a (Additional file 1: Figure S14a). These results demonstrated the PTK-7 mediated targeting ability of AuNPs-DNS to tumor cells.

The internalization pathway of AuNPs-DNS into MCF-7 cells was evaluated through flow cytometric analysis. The MCF-7 cells were firstly pre-treated with different inhibitors, such as sucrose, methyl-β-cyclodextrin, genistein, wortmannin and NaN_3_, to selectively restrain relevant internalization processes including clathrin, lipid raft, caveolae, macropinocytosis and energy dependent endocytosis, respectively. These cells were then incubated with Cy5 labeled AuNPs-DNS to measure the fluorescence intensity. The treatment with sucrose and NaN_3_ induced obvious suppressions of 43.9% and 59.5% in AuNPs-DNS_Cy5_ uptake respectively (Additional file 1: Figure S14b), illustrating that the internalization of AuNPs-DNS underwent a clathrin-dependent endocytosis pathway upon entering MCF-7 cells.

### Intracellular NIR response and anti-cancer therapy of nano-*Actinia*

**Fig. 3:**
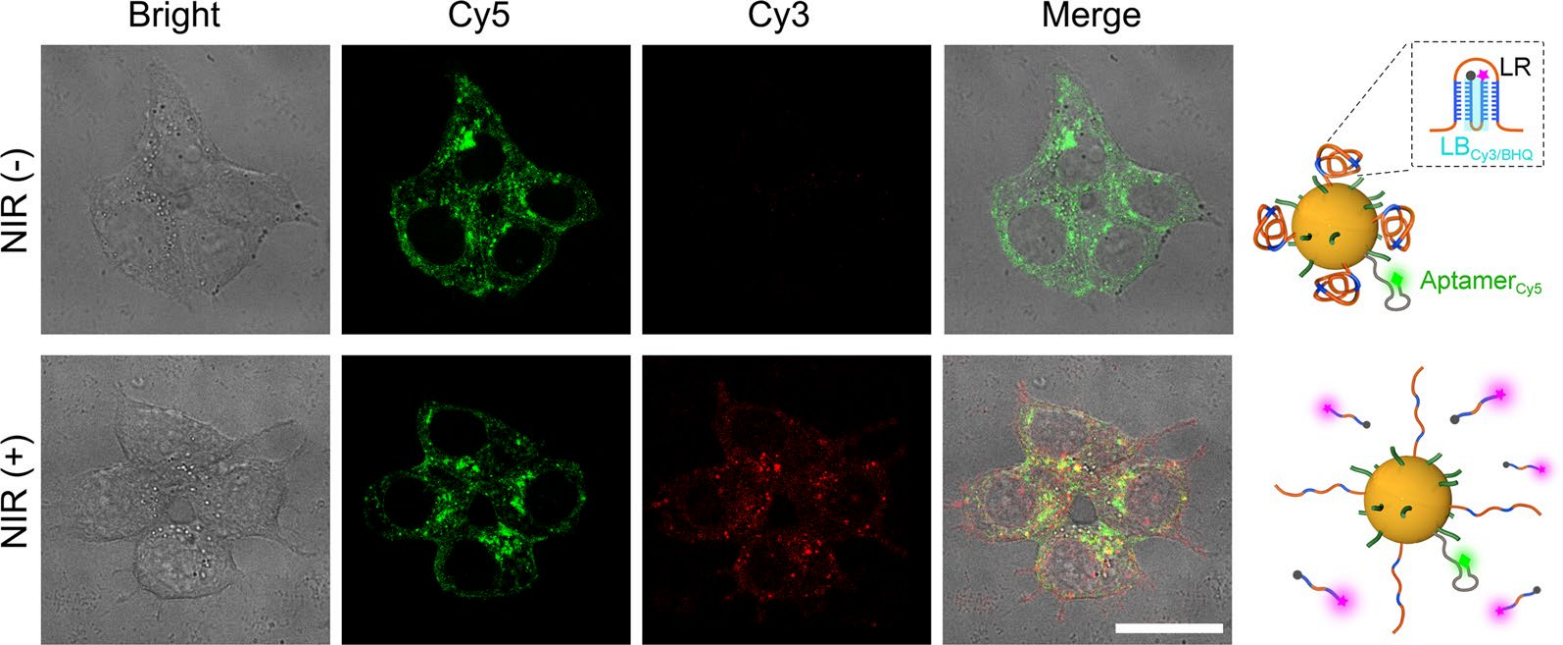
Confocal laser scanning microscopy images of MCF-7 cells treated with AuNPs-DNS_Cy5-Cy3/BHQ_ before and after NIR irradiation. The scale bar indicates 25 μm

To investigate the NIR photo-responsivity of nano-*Actinia*, MCF-7 cells were incubated with bicolor AuNPs-DNS (AuNPs-DNS_Cy5-Cy3/BHQ_) prepared using LB_Cy3/BHQ_ and Aptamer_Cy5_. In the absence of 800-nm light exposure, the incubated MCF-7 cells showed only Cy5 fluorescence due to the fluorescence quenching of Cy3 by adjacent BHQ, while the cells irradiated with 10-min NIR light exhibited bright fluorescence of both Cy5 and Cy3 (Fig. 3 and Additional file 1: Figure S15), indicating the disintegration of DNS and the liberation of LB_Cy3/BHQ_ from nanoparticles, and thus providing compelling evidence for the reliable photoresponsivity of the nano-*Actinia* and NIR light-initiated intracellular architecture collapse for the controllable drug release.

**Fig. 4:**
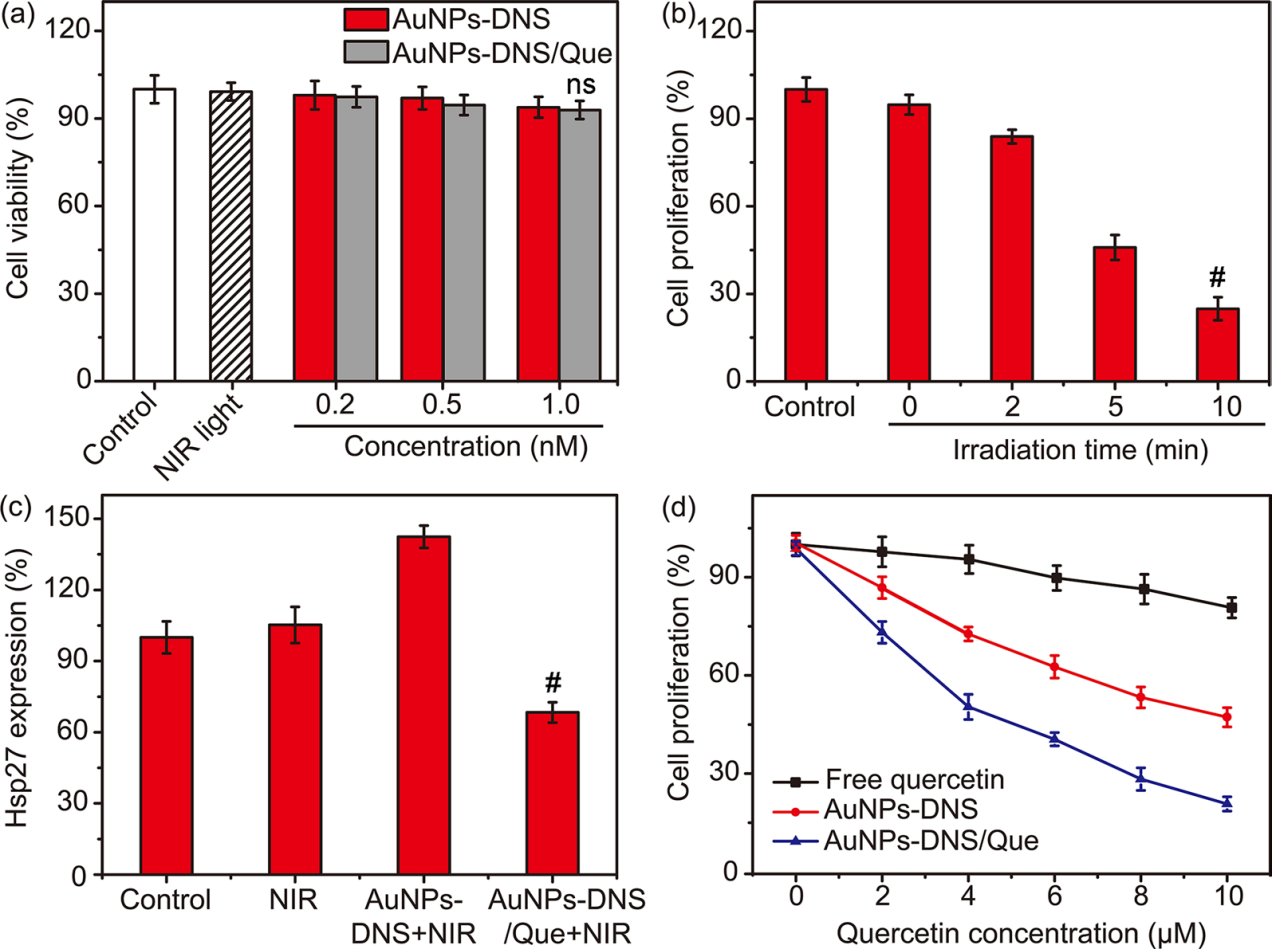
**a** Cell viability of MCF-7 cells treated with 10 min NIR light or different concentrations of AuNPs-DNS or AuNPs-DNS/Que in the absence of NIR light. Relative cell proliferation percentages of MCF-7 cells treated with **b** AuNPs-DNS/Que after NIR irradiation for different times and **d** different concentrations of quercetin, AuNPs-DNS or AuNPs-DNS/Que after 10 min NIR irradiation. **c** Relative expression levels of Hsp27 in MCF-7 cells after different treatments. (^#^*P* < 0.01) Error bars indicate means ± SD (*n* = 3)

The biocompatibility and bio-safety of AuNPs-DNS were evaluated through 3-(4,5-dimethylthiazol-2-yl)-2- diphenyltetrazolium bromide (MTT) cell cytotoxicity assay. After incubated with different concentrations of AuNPs-DNS for 6 h, the MCF-7 cells kept high viabilities over 90% in the absence of NIR light exposure (Fig. 4a). Besides, 10-min irradiation of 800-nm light (2 W cm^−2^) did not induce discernible decrease in viability rate of MCF-7 cells (Fig. 4a). These results substantiated the low cytotoxicity and good biocompatibility of the nano-*Actinia*. Moreover, the treatment of MCF-7 cells with AuNPs-DNS/Que also displayed little harm to the cells without NIR light irradiation (Fig. 4a), which further demonstrated the little leakage of quercetin during delivery and the intracellular stability as well as bio-safety of the DNA nanocarrier.

MTT assay was also used to study the cellular therapeutic efficiency of AuNPs-DNS/Que under NIR light irradiation. After incubation with AuNPs-DNS/Que and/or irradiation under 800-nm light for different times, MCF-7 cells were cultured in fresh medium containing 10% FBS for another 24 h to evaluate the cell proliferation rate. In the absence of NIR light, AuNPs-DNS/Que induced negligible influence on the cell proliferation rate, while merely 2-min NIR exposure showed the suppression of AuNPs-DNS/Que on the cell proliferation, and the suppression rate reached 75.1% at the irradiation time of 10 min (Fig. 4b), indicating the satisfactory anti-cancer therapeutic efficacy and photo-controllability of this designed *Actinia*-like DNA nanocarrier. Even at a low concentration of 0.5 nM, AuNPs-DNS/Que still represented obvious inhibition on the cell proliferation, while for normal cells (MCF-10a cells), only slight influence could be observed after treated with different concentrations of AuNPs-DNS/Que (Additional file 1: Figure S16) under NIR light irradiation, indicating the specific killing effect on tumor cells and good biocompatibility of this *Actinia*-mimetic nanocarrier.

### Enhanced therapeutic efficacy by quercetin loading

As one of the important subtype of heat shock proteins, Hsp27 is a small heat shock protein of 205 amino acids, which has been reported to play a significant role in numerous biological processes, and can modulate the response of cells to harmful stresses including heat shock [34, 35]. The abnormally elevated Hsp27 expression in tumor cells can help repairing the thermal damage to proteins and provide the tumor cells with thermal resistance [36], leading to the inadequate cell apoptosis and poor photothermal treatment efficiency. In our design, quercetin, which has been proved to be capable of suppressing the expression of Hsp27 in the previous report [26], is loaded in the double helixes of DNS to restrain the tumor thermal resistance and improve the PTT efficacy. Firstly, enzyme-linked immunosorbent assay (ELISA) was conducted to certify the downregulation of Hsp27 by AuNPs-DNS/Que. 10-min irradiation of 800-nm light did not induce obvious influence on the expression of Hsp27, while the Hsp27 level in the cells treated with AuNPs-DNS under NIR light exposure was about 1.4 times higher than that of control group with no treatment (Fig. 4c), indicating that the high temperature generated by gold nanoparticles could stimulate MCF-7 cells to upregulate Hsp27 for thermal resistance [34]. However, for the MCF-7 cells treated with AuNPs-DNS/Que after NIR irradiation, the expression of Hsp27 exhibited clear reduction in comparison with the AuNPs-DNS group (Fig. 4c), and even much lower than the control group with no treatment, which gave adequate proof for the ability of AuNPs-DNS/Que to inhibit Hsp27 expression. Although the high temperature generated by AuNPs-DNS/Que upregulated the expression of Hsp27 (Fig. 4c), the favorable delivery efficiency of this nanocomplex contributed to more powerful suppression on the expression level of Hsp27 in MCF-7 cells than free quercetin (Additional file 1: Figure S17), further indicating the favorable inhibition of AuNPs-DNS/Que on Hsp27 expression.

The strong inhibition effect of AuNPs-DNS/Que on tumor cell proliferation was further verified through treating MCF-7 cells with AuNPs-DNS/Que at various concentrations of quercetin for 6 h and 800-nm light irradiation for 10 min, and then culturing for another 24 h to conduct MTT assay. In the case of low doses of quercetin, the proliferation of MCF-7 cells did not exhibit noticeable decrease for all groups (Fig. 4d). As the concentration of quercetin increased, the incubation of AuNPs-DNS/Que induced a conspicuous falling-off on the cell proliferation rate, while higher administration dosage was demanded for AuNPs-DNS to achieve equivalent inhibition level of cell proliferation (Fig. 4d), indicating the elevated therapeutic efficacy attributable to the loaded quercetin. Additionally, flow cytometric assay was also conducted to demonstrate the superior anti-cancer ability of AuNPs-DNS/Que. The MCF-7 cells were treated with different formulations (quercetin, AuNPs-DNS or AuNPs-DNS/Que) for 6 h and then10-min NIR light irradiation. After incubation in fresh culture medium for another 24 h and then stained with the Annexin V-fluorescein isothiocyanate (FITC)/propidium iodide (PI) cell apoptotic kit, the cell apoptosis rate was examined through flow cytometry, which presented the highest tumor cell killing efficacy of AuNPs-DNS/Que under NIR light exposure (Additional file 1: Figure S18). In the absence of 800-nm light irradiation, negligible apoptosis occurred for cells treated with AuNPs-DNS or AuNPs-DNS/Que (Additional file 1: Figure S18c and d), illustrating the ideal photo-controllability for drug release and anti-cancer therapy of this DNA nanocarrier.

### In vivo anti-cancer therapy

**Fig. 5:**
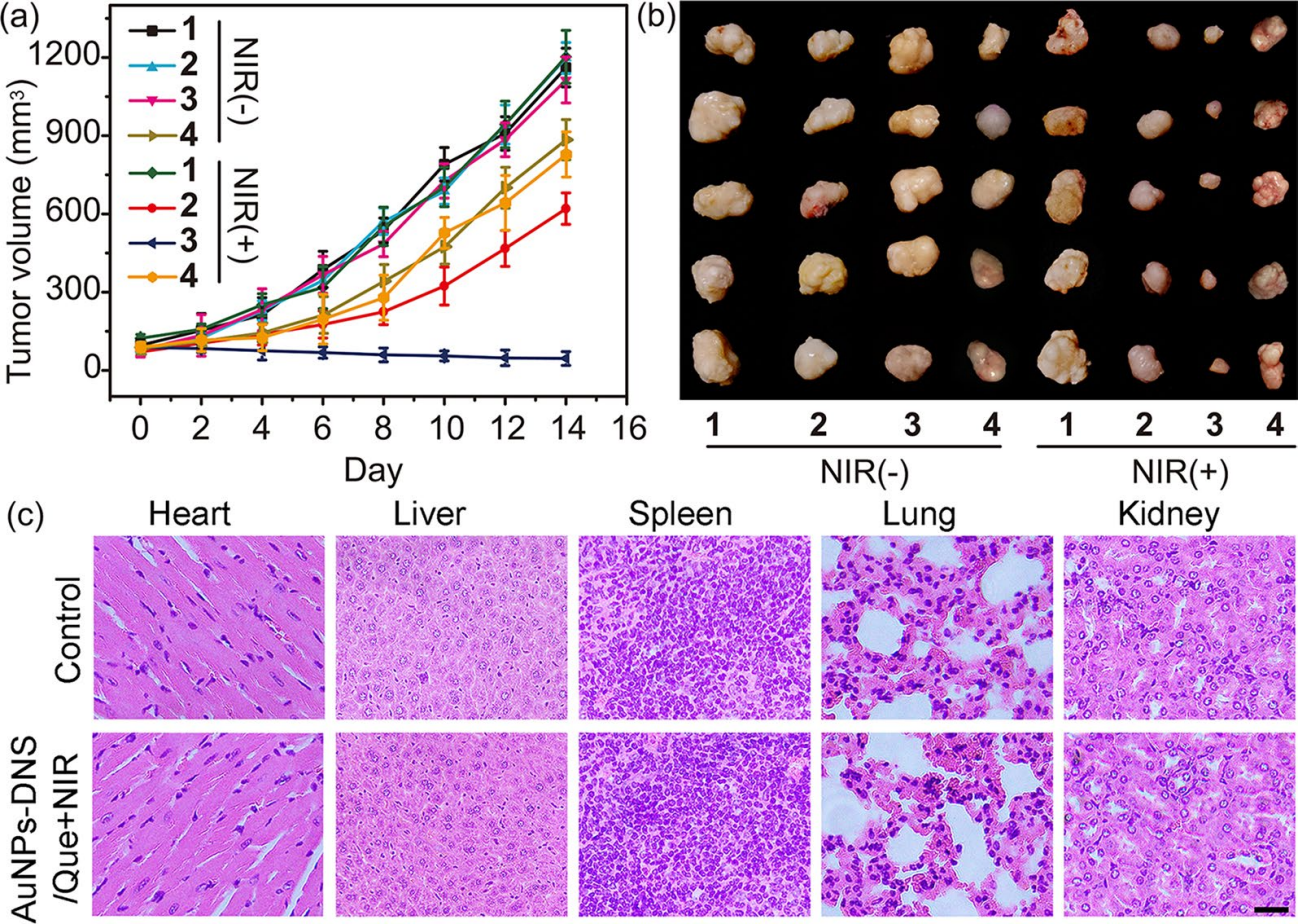
**a** Change of tumor volumes and **b** representative tumor images of mice treated with saline (1), AuNPs-DNS (2), AuNPs-DNS/Que (3) or free quercetin (4) in the absence or presence of 800-nm light irradiation. Error bars indicate means ± SD (*n* = 5). **c** Histological observations of the major organs from mice treated with saline or AuNPs-DNS/Que after NIR irradiation. The scale bar indicates 100 μm

The in vivo anti-cancer therapy of AuNPs-DNS/Que was clarified using mice bearing MCF-7 xenograft tumors. Firstly, the in vivo photothermal effect of AuNPs-DNS/Que was evaluated through monitoring the temperature change at the tumor site of mice injected with AuNPs-DNS/Que under NIR light, which showed much higher temperature at tumor site (45.4 ºC) after 10-min irradiation in comparison with the mice treated with saline (29.7 ºC) (Additional file 1: Figure S19). For the in vivo anti-cancer treatment experiment, when the tumors grew up to 80 mm^3^, the mice were randomly grouped and intratumorally injected with saline, free quercetin, AuNPs-DNS or AuNPs-DNS/Que. Then the tumors in different group of mice were irradiated with 800-nm laser (1 W cm^−2^) for 10 min or not. The treatment was repeated every 3 days, and the variation of tumor volumes was recorded to investigate the inhibition capacity of AuNPs-DNS/Que for tumor growth. In the presence of NIR light irradiation, AuNPs-DNS/Que presented the highest anti-cancer therapy efficacy and obviously prevented the proliferation of tumor compared to free quercetin or AuNPs-DNS without quercetin loading (Fig. 5a, b and Additional file 1: Figure S20), which demonstrated the promotion of quercetin for the photothermal therapy efficacy and the superior anti-cancer ability of this DNA nanocarrier. For the mice without NIR light exposure, the curve of tumor volume after treatment of AuNPs-DNS/Que demonstrated a similar tendency in comparison with the control group treated with saline, indicating the desirable photo-controllability of AuNPs-DNS/Que for the drug release as well as anti-cancer treatment. Additionally, the tumor tissues of different groups were collected at DAY 14 and stained with hematoxylin and eosin (HE) for the histological analysis, which indicated the same conclusion and exhibited massive cell remission and the highest cell killing efficacy in the mice treated with AuNPs-DNS/Que under NIR exposure (Additional file 1: Figure S21). The biodistribution analysis of Cy5 labeled AuNPs-DNS exhibited its good accumulation into tumor sites at 6 h after injection (Additional file 1: Figure S22), which demonstrated the brilliant targeting capacity of AuNPs-DNS to tumors in vivo. During the 14-day experiment, no apparent reduction in body weight was observed in all of the mice (Additional file 1: Figure S23), and the histological analysis of the heart, liver, spleen, lung and kidney suggested no pathological abnormality even for the mice treated with AuNPs-DNS/Que in the presence of NIR light (Fig. 5c). These results illustrated the satisfactory biocompatibility and specificity of this *Actinia*-like DNA nanocarrier.

## Conclusion

This work designs an *Actinia*-like AuNPs-DNA complex for controllable drug release and enhanced photothermal therapy. The collapsible DNA architecture on the AuNPs core guarantees the high drug-loading capacity as well as sensitive response to the local high temperature induced by the photothermal effect of AuNPs. The compressed DNA nanosphere close to the AuNPs can rapidly disassemble under 800-nm light exposure to achieve efficient drug release of 87.6% in merely 10 min. The in vitro and in vivo experiments clarify obvious inhibition efficiency to cell proliferation and satisfactory therapeutic efficacy towards tumors in mice. We envision that this biomimetic DNA-AuNP nanocarrier might provide impressive contribution for the development of on-demand drug delivery systems and anti-cancer clinical therapy.

## Supplementary Information

**Additional file 1: Table S1.** Sequences of all oligonucleotides used in this work. **Figure S1.** PAGE analysis of circular DNA template synthesis. Lanes 1–3 represent ligation DNA, phosphorylated linear DNA and the circular DNA product respectively. **Figure S2.** Dynamic light scattering (DLS) analysis of the compressed DNA nanosphere. **Figure S3.** (a) Zeta potentials of AuNPs, AuNPs-primer and AuNPs-LR. The data error bars indicate means ± SD (*n* = 3). (b) UV–Vis absorption spectrum of AuNPs-primer. **Figure S4.** DLS analysis of AuNPs, AuNPs-primer, AuNPs-LR and AuNPs-DNS. **Figure S5.** Hydration diameters of AuNPs-LR and AuNPs-DNS through synthesis with (1) 50 nM DNA primer, 550 nM DNA spacer and 100 nM Sgc8 aptamer, (2) 100 nM DNA primer, 500 nM DNA spacer and 100 nM Sgc8 aptamer and (3) 200 nM DNA primer, 400 nM DNA spacer and 100 nM Sgc8 aptamer. **Figure S6.** Calibration curves for (a) FAM labeled DNA primer, (c) Cy3 labeled DNA spacer and (e) Cy5-labeled Sgc8 aptamer, and fluorescence spectra of supernatants after incubation with AuNPs at λ_ex_ of (b) 494 nm (FAM excitation), (d) 510 nm (Cy3 excitation) and (f) 649 nm (Cy5 excitation). The data error bars indicate means ± SD (*n* = 3). **Figure S7.** Temperature variation of 500 μL PBS or AuNPs-DNS solution with different concentrations under 800 nm light. **Figure S8.** DLS analysis of AuNPs-DNS after 10-min NIR irradiation. **Figure S9.** (a) Fluorescence spectra of quercetin and the supernatant after quercetin loading. (b) Calibration curve for quercetin. The data error bars indicate means ± SD (*n* = 3). **Figure S10.** TEM images of AuNPs-DNS and AuNPs-DNS/Que. (scale bar: 100 nm). **Figure S11.** Release percentages of quercetin from AuNPs-DNS/Que with temperature under 800-nm light exposure. **Figure S12.** (a) Schematic illustration and (b) DLS analysis of control nanoparticles synthesized with single strands (control ssDNA) instead of LB. (c) Release percentages of quercetin from the control nanoparticles under 800-nm light exposure. The data error bars indicate means ± SD (*n* = 3). **Figure S13.** (a) Variation of hydration diameter of AuNPs-DNS and (b) quercetin leakage from AuNPs-DNS/Que after incubation in PBS and PBS containing 10% FBS for different times. The data error bars indicate means ± SD (*n* = 3). **Figure S14.** (a) Flow cytometric assay of MCF-7 cells incubated with AuNPs-DNS or AuNPs-DNS_Control_ and MCF-10a cells incubated with AuNPs-DNS. (b) Uptake rates of AuNPs-DNS in MCF-7 cells and MCF-7 cells preincubated with different inhibitors. The data error bars indicate means ± SD (*n* = 3). **Figure S15.** Fluorescence intensities in confocal laser scanning microscopy images of MCF-7 cells treated with AuNPs-DNS_Cy5-Cy3/BHQ_ before and after NIR irradiation. **Figure S16.** Relative cell proliferation percentages of MCF-7 cells and MCF-10a cells treated with different concentrations of AuNPs-DNS/Que after 10-min NIR irradiation. Error bars indicate means ± SD (*n* = 3). **Figure S17.** Relative expression levels of Hsp27 in MCF-7 cells treated with different concentrations of AuNPs-DNS/Que or free quercetin under 10-min NIR irradiation. Error bars indicate means ± SD (*n* = 3). **Figure S18.** Flow cytometric assays of (a) and (e) MCF-7 cells, and MCF-7 cells incubated with (b) and (f) quercetin, (c) and (g) AuNPs-DNS or (d) and (h) AuNPs-DNS/Que in the (a-d) absence or (e–h) presence of 10-min NIR light irradiation. **Figure S19.** Thermographic images of mice treated with PBS or. **Figure S20.** Representative images of mice treated with saline, AuNPs-DNS, AuNPs-DNS/Que or free quercetin with or without 800-nm light irradiation at Day 14. **Figure S21.** Histological observations of tumor tissues after treatments with saline, AuNPs-DNS, AuNPs-DNS/Que or free quercetin in the absence or presence of NIR light exposure. The scale bar indicates 100 μm. **Figure S22.** In vivo fluorescence imaging of mice treated with PBS or AuNPs-DNS_Cy5_. **Figure S23.** Changes in body weight of mice treated with saline (1), AuNPs-DNS (2), AuNPs-DNS/Que (3) or free quercetin (4) with or without 800-nm light irradiation. The data error bars indicate means ± SD (*n* = 5).

## Data Availability

The data that support the findings of this study are available from the corresponding authors upon reasonable request.

## Abbreviations

AuNP: Gold nanoparticle
NIR: Near-infrared
LR: Long single DNA strands
RCA: Rolling circle amplification
LB: Bundling DNA strands
DNS: DNA nanosphere
Que: Quercetin
Hsp27: Heat shock protein 27
PTT: Photothermal therapy
PAGE: Polyacrylamide gel electrophoresis
MTT: 3-(4,5-Dimethylthiazol-2-yl)-2-diphenyltetrazolium bromide
ELISA: Enzyme-linked immunosorbent assay
SDS: Sodium dodecyl sulfate
TCEP: (2-Carboxyethyl) phosphine hydrochloride
HE: Hematoxylin–eosin
DLS: Dynamic light scattering
TEM: Transmission electron microscopic
CLSM: Confocal laser scanning microscope
