# Supplementary material for: Biomimetic retractable DNA nanocarrier with sensitive responsivity for efficient drug delivery and enhanced photothermal therapy
Source: J Nanobiotechnology. 2023 Feb 9;21:46. doi: 10.1186/s12951-023-01806-5 (PMC9909879; doi:10.1186/s12951-023-01806-5)
Supplement: Supplementary file 1 — Additional file 1: Table S1. Sequences of all oligonucleotides used in this work. Figure S1. PAGE analysis of circular DNA template synthesis. Lanes 1–3 represent ligation DNA, phosphorylated linear DNA and the circular DNA product respectively. Figure S2. Dynamic light scattering (DLS) analysis of the compressed DNA nanosphere. Figure S3. (a) Zeta potentials of AuNPs, AuNPs-primer and AuNPs-LR. The data error bars indicate means ± SD (n = 3). (b) UV–Vis absorption spectrum of AuNPs-primer. Figure S4. DLS analysis of AuNPs, AuNPs-primer, AuNPs-LR and AuNPs-DNS. Figure S5. Hydration diameters of AuNPs-LR and AuNPs-DNS through synthesis with (1) 50 nM DNA primer, 550 nM DNA spacer and 100 nM Sgc8 aptamer, (2) 100 nM DNA primer, 500 nM DNA spacer and 100 nM Sgc8 aptamer and (3) 200 nM DNA primer, 400 nM DNA spacer and 100 nM Sgc8 aptamer. Figure S6. Calibration curves for (a) FAM labeled DNA primer, (c) Cy3 labeled DNA spacer and (e) Cy5-labeled Sgc8 aptamer, and fluorescence spectra of supernatants after incubation with AuNPs at λex of (b) 494 nm (FAM excitation), (d) 510 nm (Cy3 excitation) and (f) 649 nm (Cy5 excitation). The data error bars indicate means ± SD (n = 3). Figure S7. Temperature variation of 500 μL PBS or AuNPs-DNS solution with different concentrations under 800 nm light. Figure S8. DLS analysis of AuNPs-DNS after 10-min NIR irradiation. Figure S9. (a) Fluorescence spectra of quercetin and the supernatant after quercetin loading. (b) Calibration curve for quercetin. The data error bars indicate means ± SD (n = 3). Figure S10. TEM images of AuNPs-DNS and AuNPs-DNS/Que. (scale bar: 100 nm). Figure S11. Release percentages of quercetin from AuNPs-DNS/Que with temperature under 800-nm light exposure. Figure S12. (a) Schematic illustration and (b) DLS analysis of control nanoparticles synthesized with single strands (control ssDNA) instead of LB. (c) Release percentages of quercetin from the control nanoparticles under 800-nm light exposure. The data error [file 12951_2023_1806_MOESM1_ESM.doc]

**Additional file**

**Biomimetic retractable DNA nanocarrier with sensitive responsivity for efficient drug delivery and enhanced photothermal therapy**

Yuanhuan Yang^1^, Xueting Cai^2^, Menglin Shi^1^, Xiaobo Zhang^3^, Yang Pan^1^, Yue Zhang^1^*, Huangxian Ju^3^*, and Peng Cao^1,2,4^*

^1^ School of Pharmacy, Nanjing University of Chinese Medicine, Nanjing 210023, China.

^2^ Affiliated Hospital of Integrated Traditional Chinese and Western Medicine, Nanjing University of Chinese Medicine, Nanjing 210028, China.

^3^ State Key Laboratory of Analytical Chemistry for Life Science, School of Chemistry and Chemical Engineering, Nanjing University, Nanjing 210023, China.

^4^ Zhenjiang Hospital of Chinese Traditional and Western Medicine, Zhenjiang 212002, China.

Corresponding authors: Yue Zhang: zhangyue035@njucm.edu.cn; Huangxian Ju: hxju@nju.edu.cn; Peng Cao: cao_peng@njucm.edu.cn

**Table S1.** Sequences of all oligonucleotides used in this work.

| Oligonucleotides | Oligonucleotides sequences |
| --- | --- |
| Ligation DNA | 5'-TGCTTTAGGAGCTGTAAACG-3' |
| Phosphorylated linear DNA | 5'-P-TCCTAAAGCATGACCTTCCGATGT*CGGTGAGACGTTTACAGC*-3' |
| DNA primer | 5'-SH-TTTTTTTTTTTTTTT*GCTGTAAACGTCTCACCG*-3' |
| Primer_FAM_ | 5'-SH-TTTTTTTTTTTTTTT*GCTGTAAACGTCTCACCG*-FAM-3' |
| Bundling strand LB | 5'-GGAAGGTCATAGCCGTCTCACCGA-3' |
| LB_Cy3/BHQ_ | 5'-Cy3-GGAAGGTCATAGCCGTCTCACCGA-BHQ-3' |
| Control ssDNA | 5'-GTCTCACCGACATCGGAAGGTCAT-3' |
| Sgc8 aptamer | 5'-SH-TTTTTTTTTTTTTTTTTTTTCACTACAGAGGTTGCGTCTGTCC CACGTTGTCAT GGGGGGTTGGCCTG-3' |
| Aptamer_Cy5_ | 5'-SH-TTTTTTTTTTTTTTTTTTTTCACTACAGAGGTTGCGTCTGTCC  CACGTTGTCAT GGGGGGTTGGCCTG-Cy5-3' |
| Control_Cy5_ | 5'-SH-TTTTTTTTTTTTTTTTTTTTGATGCAATTCCACTAATCAACCGTC CAAATTTCA GGTGATTACTGCAT-Cy5-3' |
| DNA spacer | 5'-SH-TTTTTTTTTTTTTTTTTTTT-3' |
| Spacer_Cy3_ | 5'-SH-TTTTTTTTTTTTTTTTTTTT-Cy3-3' |

The binding regions between phosphorylated linear DNA and ligation DNA as well as DNA primer were shown in underlined and italics respectively. The hybridization parts of phosphorylated linear DNA with bundling strand LB are labeled in the same color.

**
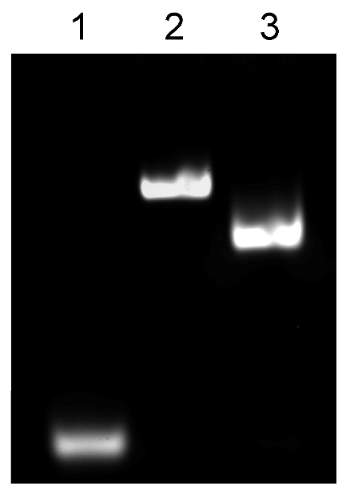
**

**Figure S1.** PAGE analysis of circular DNA template synthesis. Lanes 1-3 represent ligation DNA, phosphorylated linear DNA and the circular DNA product respectively.

**
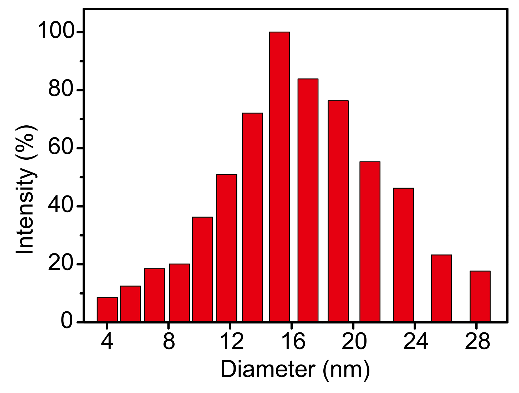
**

**Figure S2.** Dynamic light scattering (DLS) analysis of the compressed DNA nanosphere.

**
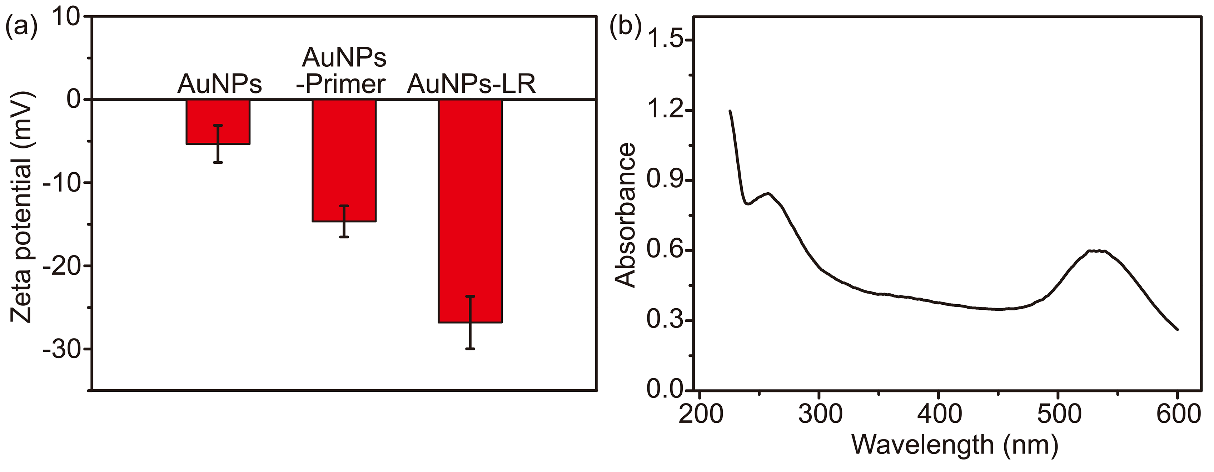
**

**Figure S3.** (a) Zeta potentials of AuNPs, AuNPs-primer and AuNPs-LR. The data error bars indicate means ± SD (*n* = 3). (b) UV-Vis absorption spectrum of AuNPs-primer.

**
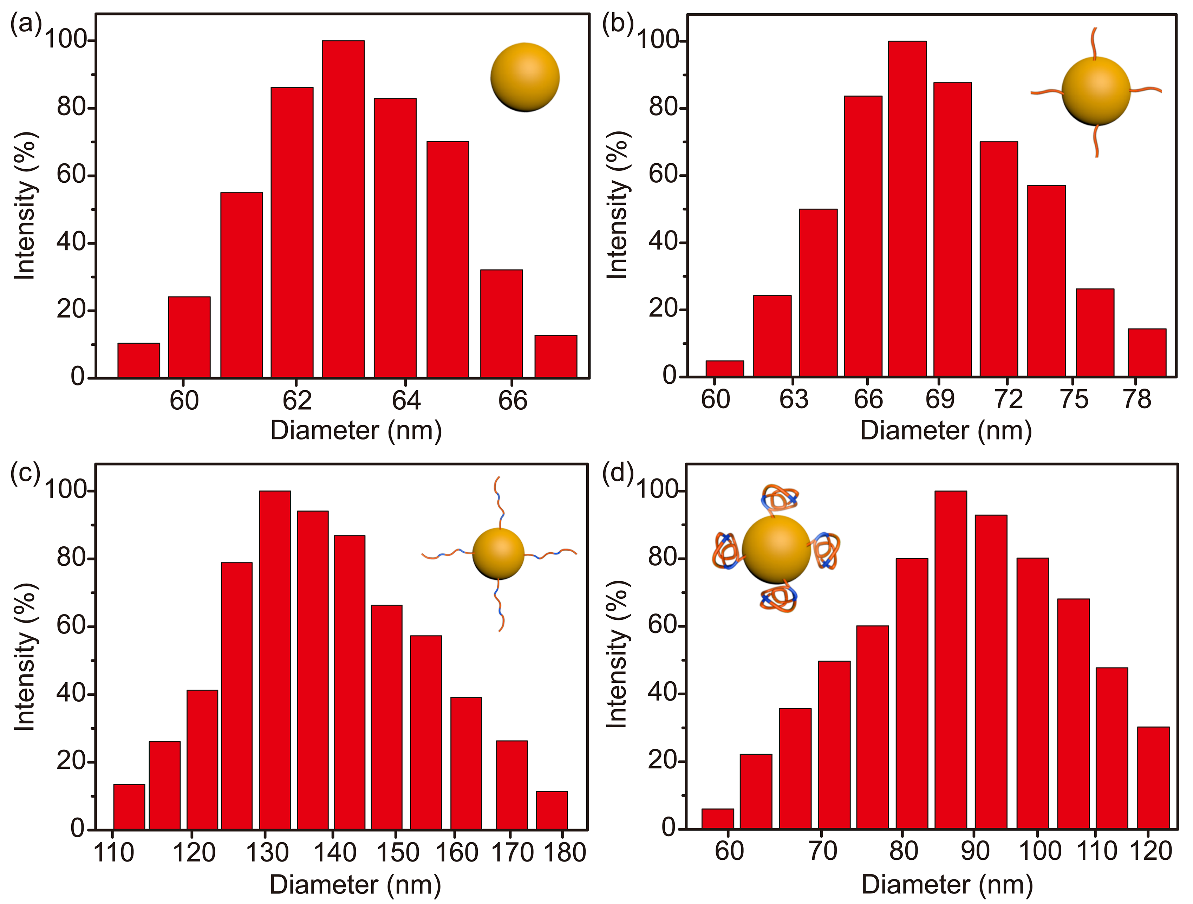
**

**Figure S4.** DLS analysis of AuNPs, AuNPs-primer, AuNPs-LR and AuNPs-DNS.


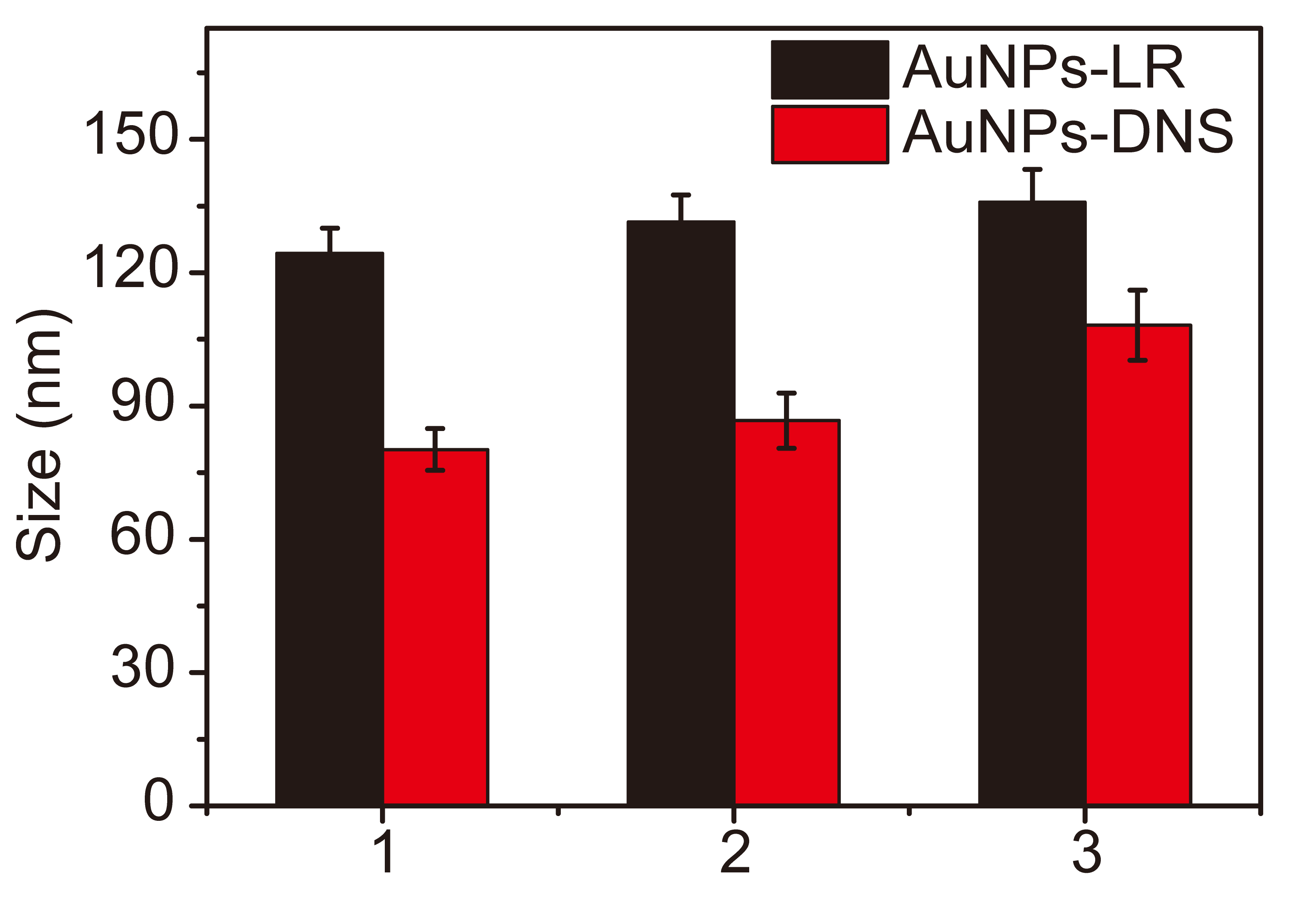


**Figure S5.** Hydration diameters of AuNPs-LR and AuNPs-DNS through synthesis with (1) 50 nM DNA primer, 550 nM DNA spacer and 100 nM Sgc8 aptamer, (2) 100 nM DNA primer, 500 nM DNA spacer and 100 nM Sgc8 aptamer and (3) 200 nM DNA primer, 400 nM DNA spacer and 100 nM Sgc8 aptamer.

**
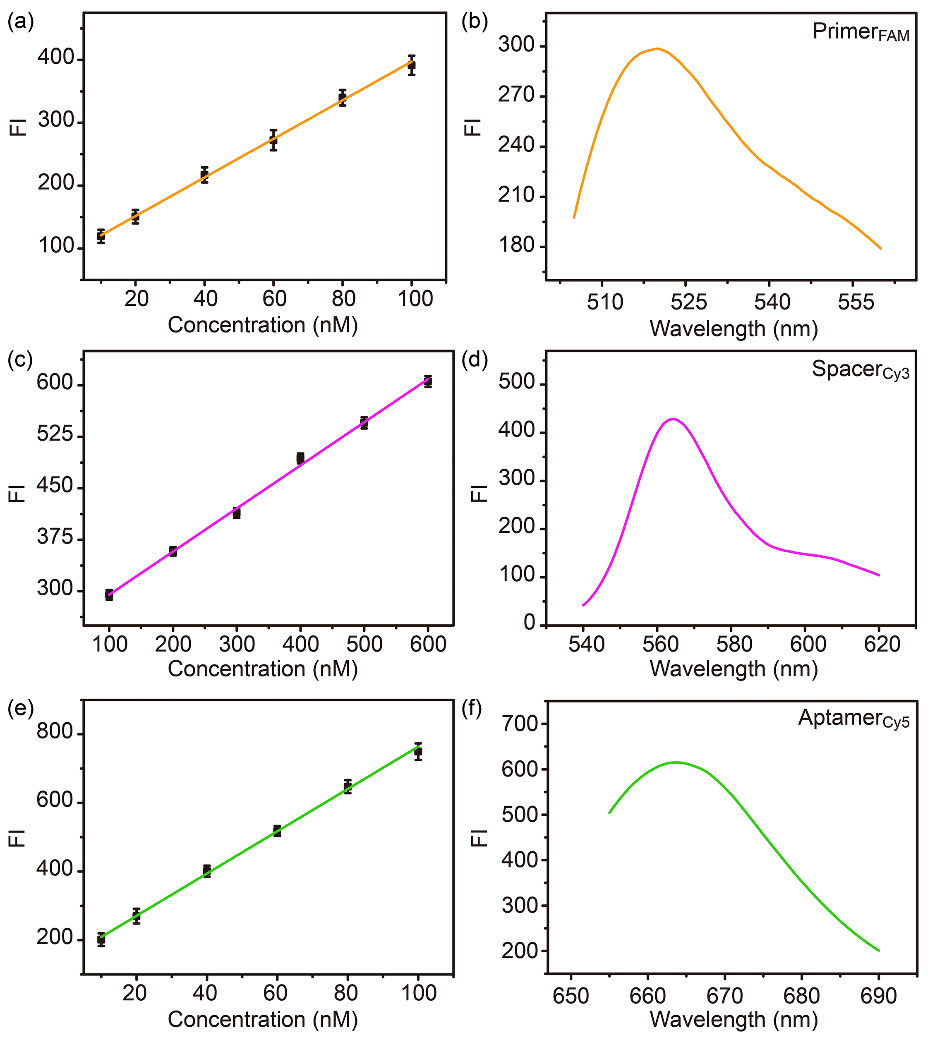
**

**Figure S6.** Calibration curves for (a) FAM labeled DNA primer, (c) Cy3 labeled DNA spacer and (e) Cy5-labeled Sgc8 aptamer, and fluorescence spectra of supernatants after incubation with AuNPs at λ_ex_ of (b) 494 nm (FAM excitation), (d) 510 nm (Cy3 excitation) and (f) 649 nm (Cy5 excitation). The data error bars indicate means ± SD (*n* = 3).

**
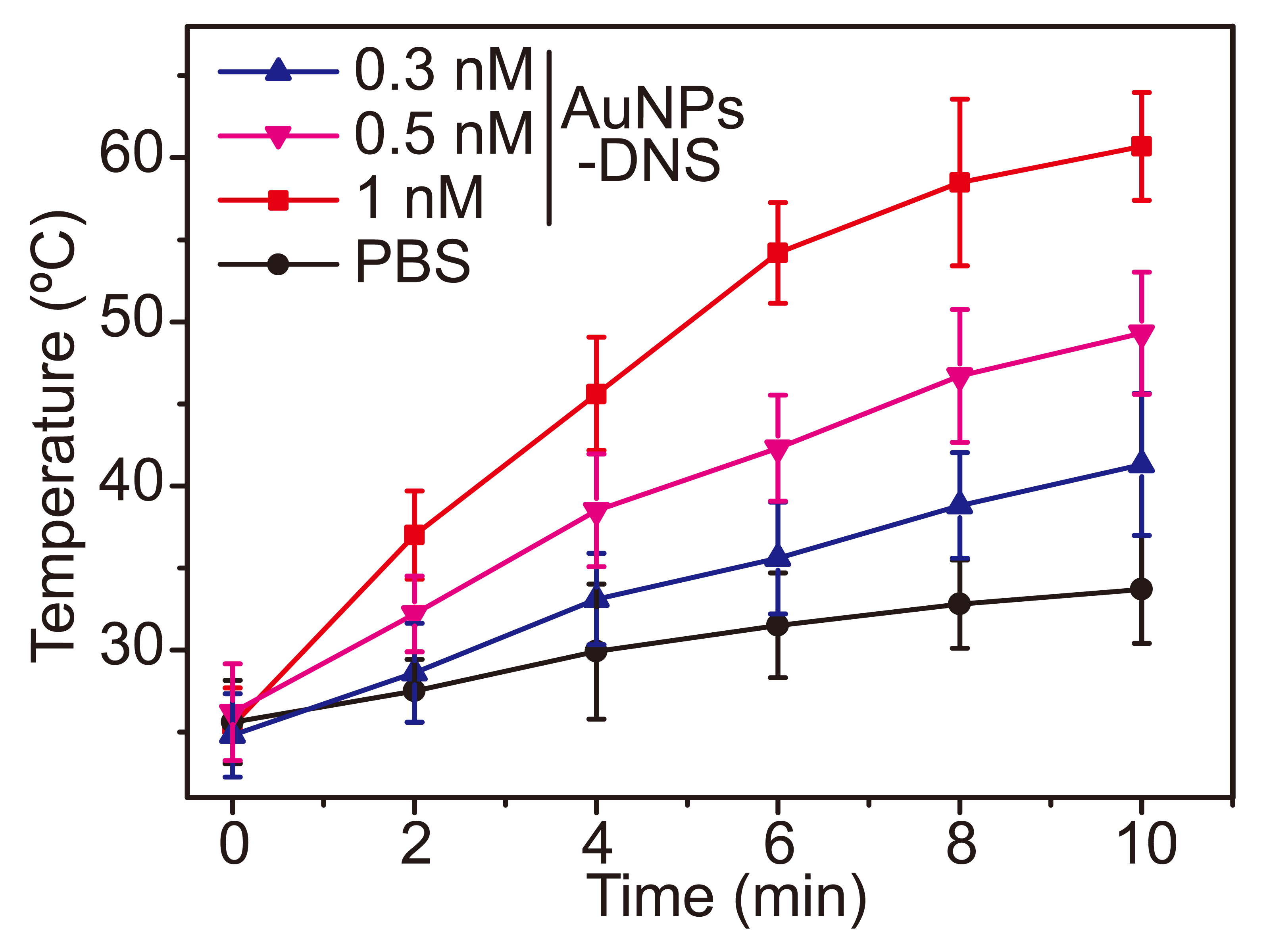
**

**Figure S7.** Temperature variation of 500 μL PBS or AuNPs-DNS solution with different concentrations under 800 nm light.

**
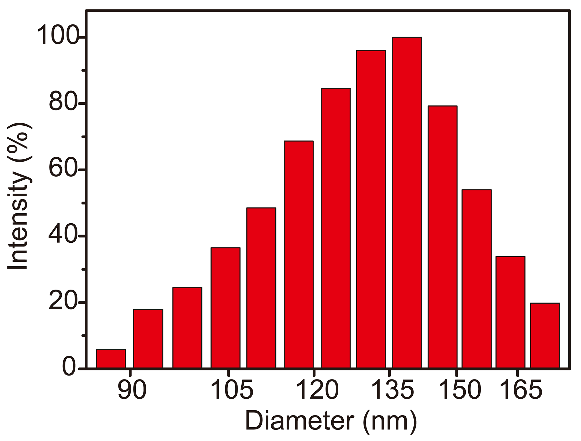
**

**Figure S8.** DLS analysis of AuNPs-DNS after 10-min NIR irradiation.

**
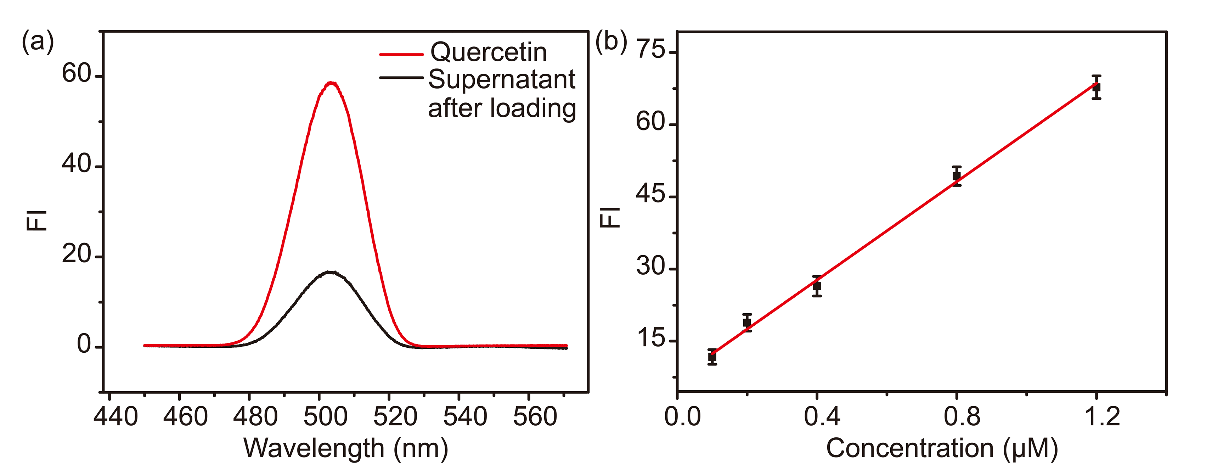
**

**Figure S9.** (a) Fluorescence spectra of quercetin and the supernatant after quercetin loading. (b) Calibration curve for quercetin. The data error bars indicate means ± SD (*n* = 3).


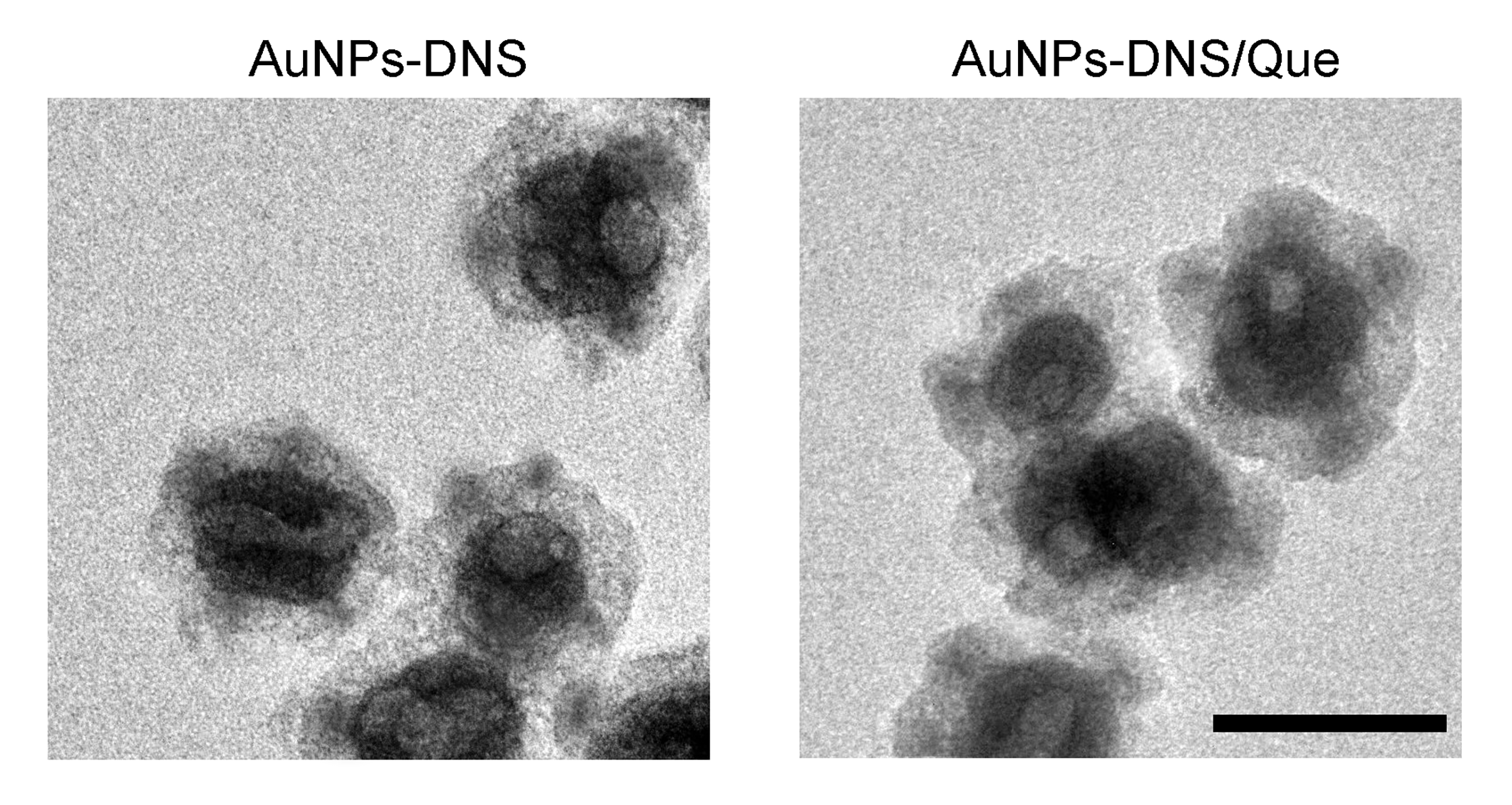


**Figure S10.** TEM images of AuNPs-DNS and AuNPs-DNS/Que. (scale bar: 100 nm)


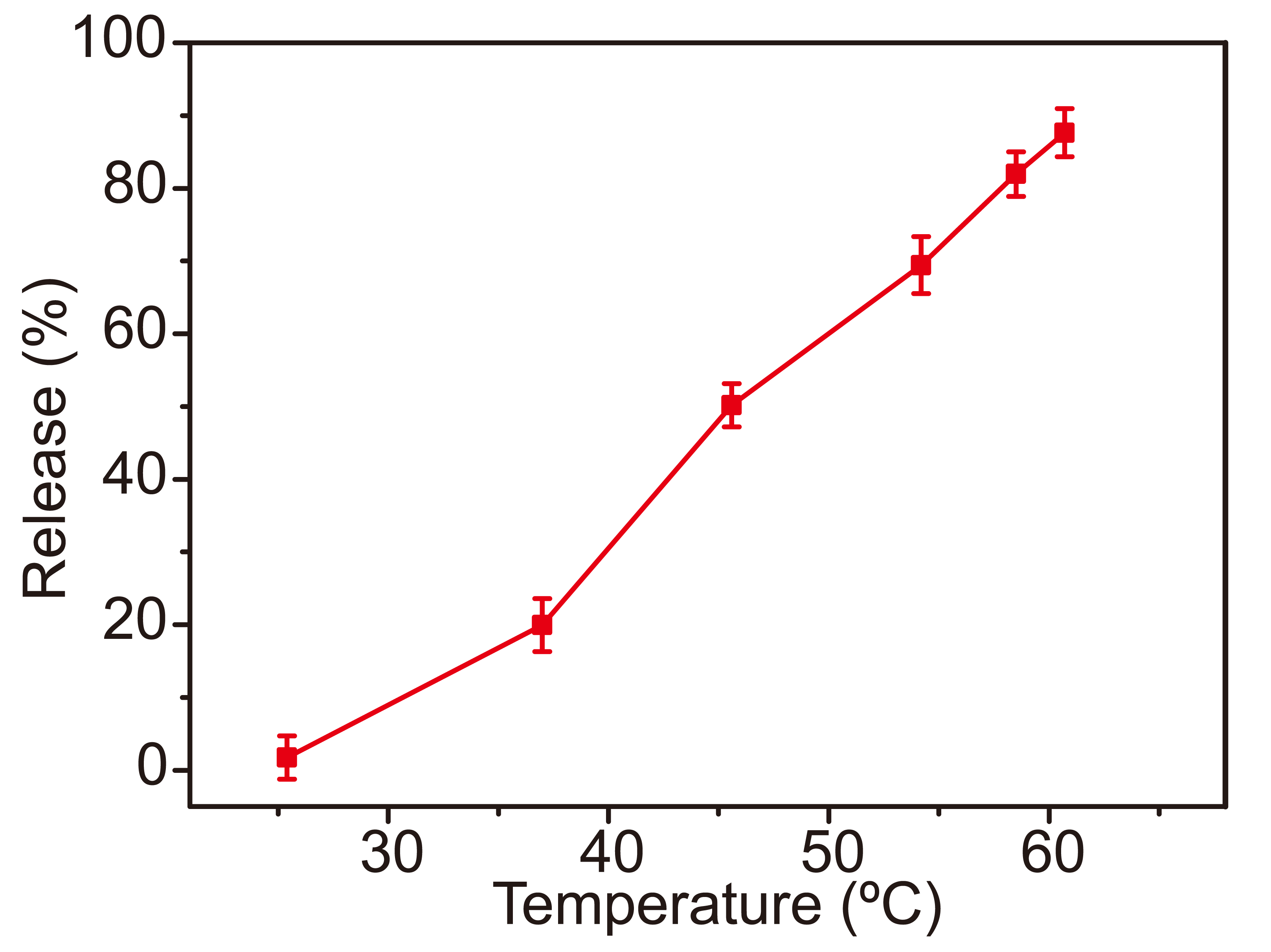


**Figure S11.** Release percentages of quercetin from AuNPs-DNS/Que with temperature under 800-nm light exposure.


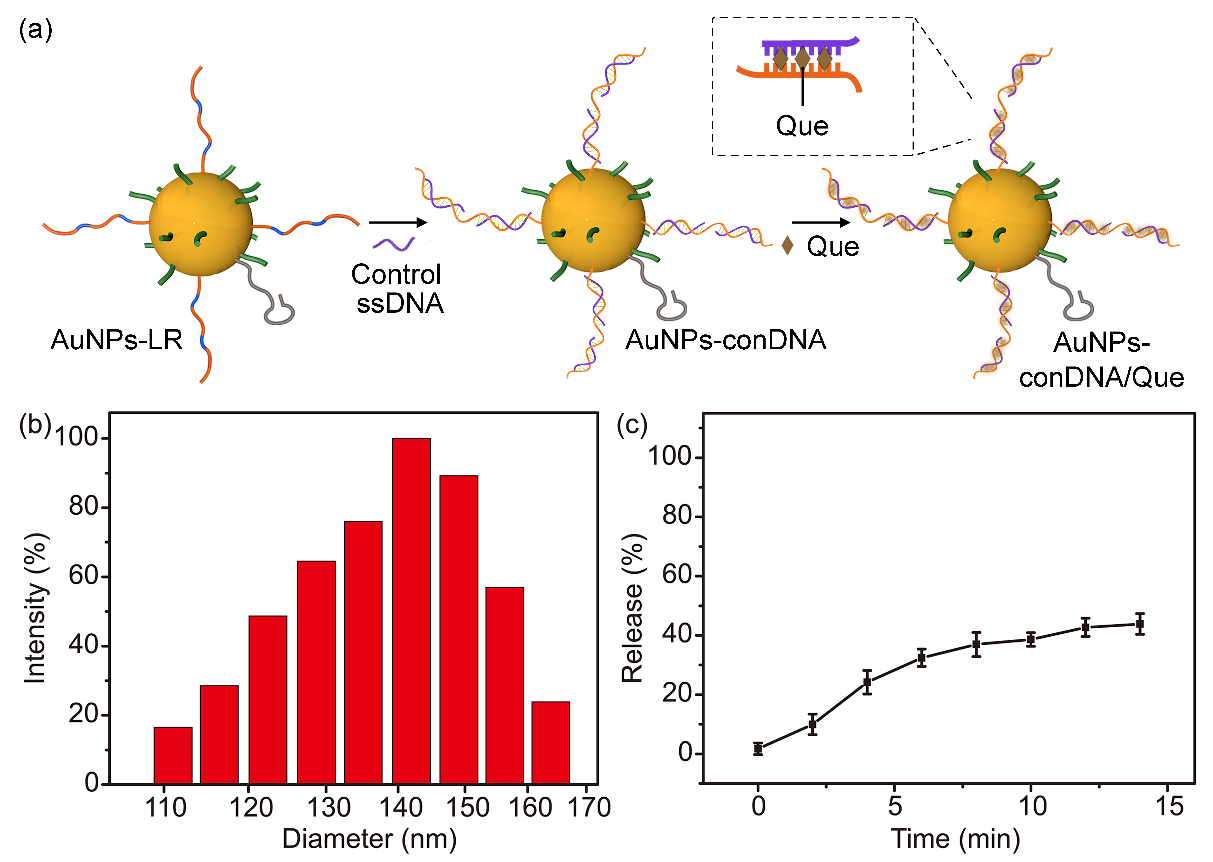


**Figure S12.** (a) Schematic illustration and (b) DLS analysis of control nanoparticles synthesized with single strands (control ssDNA) instead of LB. (c) Release percentages of quercetin from the control nanoparticles under 800-nm light exposure. The data error bars indicate means ± SD (*n* = 3).

**
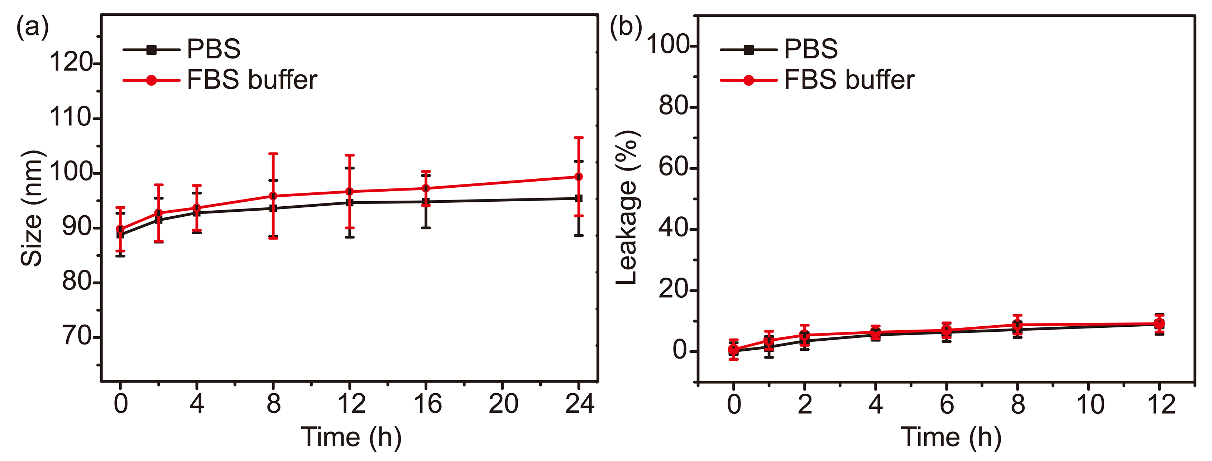
**

**Figure S13.** (a) Variation of hydration diameter of AuNPs-DNS and (b) quercetin leakage from AuNPs-DNS/Que after incubation in PBS and PBS containing 10% FBS for different times. The data error bars indicate means ± SD (*n* = 3).

**
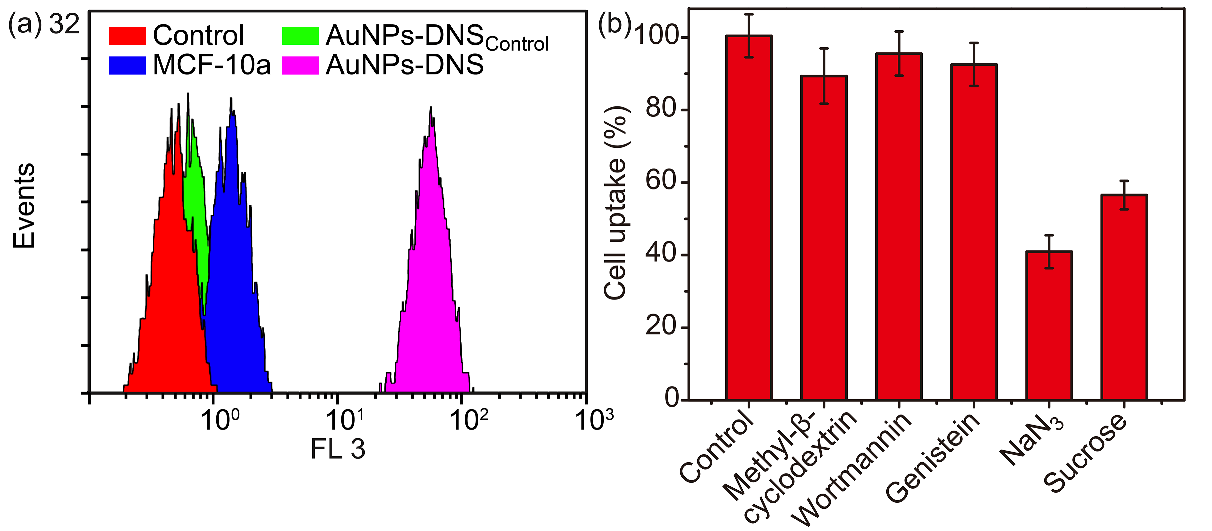
**

**Figure S14.** (a) Flow cytometric assay of MCF-7 cells incubated with AuNPs-DNS or AuNPs-DNS_Control_ and MCF-10a cells incubated with AuNPs-DNS. (b) Uptake rates of AuNPs-DNS in MCF-7 cells and MCF-7 cells preincubated with different inhibitors. The data error bars indicate means ± SD (*n* = 3).


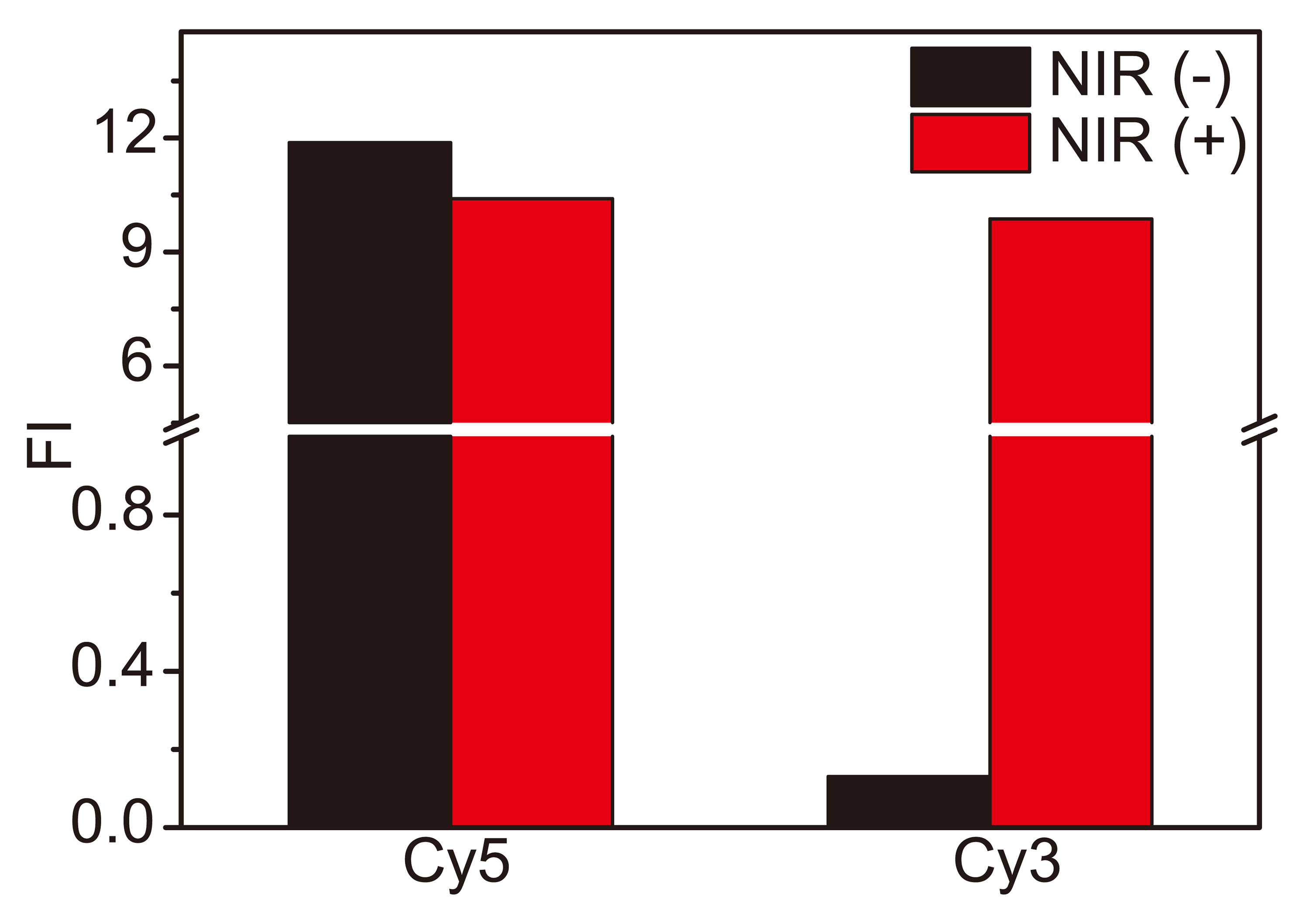


**Figure S15.** Fluorescence intensities in confocal laser scanning microscopy images of MCF-7 cells treated with AuNPs-DNS_Cy5-Cy3/BHQ_ before and after NIR irradiation.


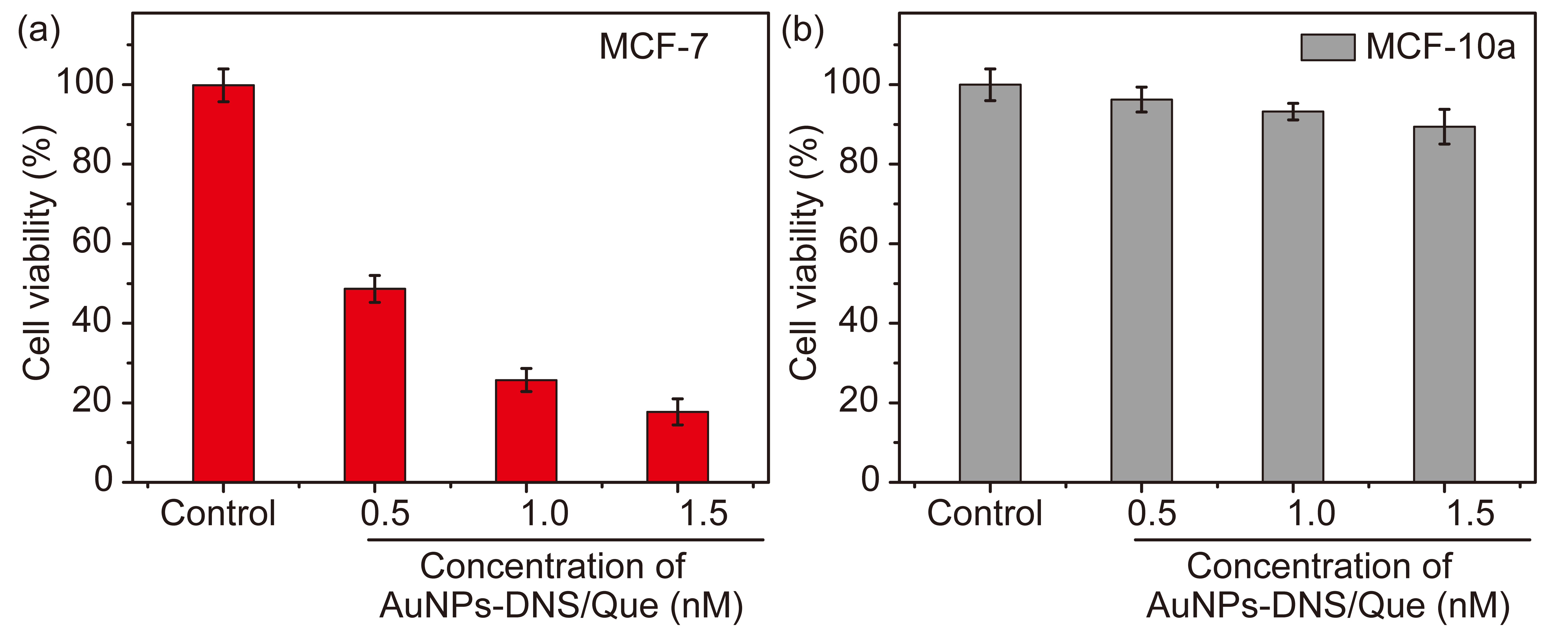


**Figure S16.** Relative cell proliferation percentages of MCF-7 cells and MCF-10a cells treated with different concentrations of AuNPs-DNS/Que after 10-min NIR irradiation. Error bars indicate means ± SD (*n* = 3)


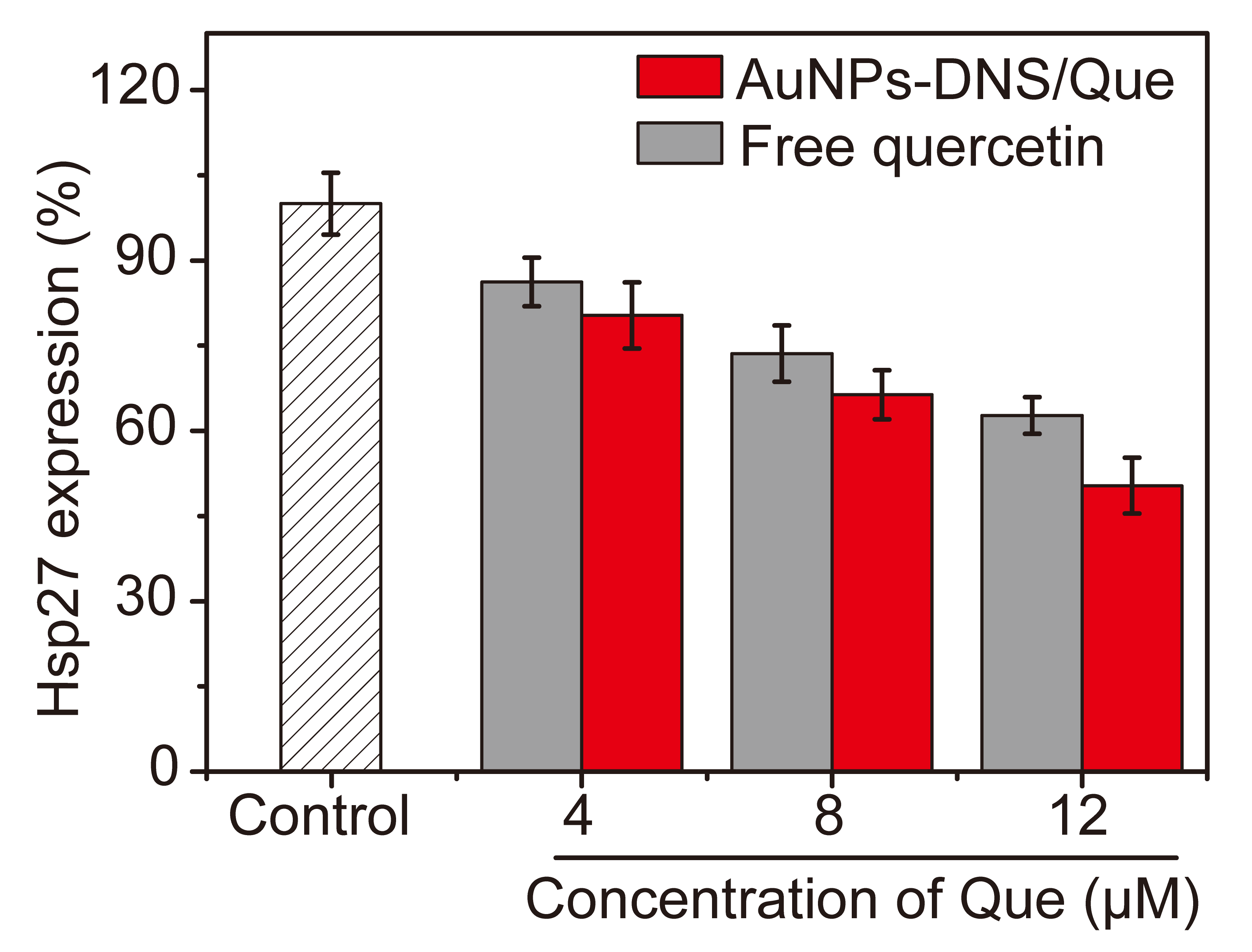


**Figure S17.** Relative expression levels of Hsp27 in MCF-7 cells treated with different concentrations of AuNPs-DNS/Que or free quercetin under 10-min NIR irradiation. Error bars indicate means ± SD (*n* = 3)

**
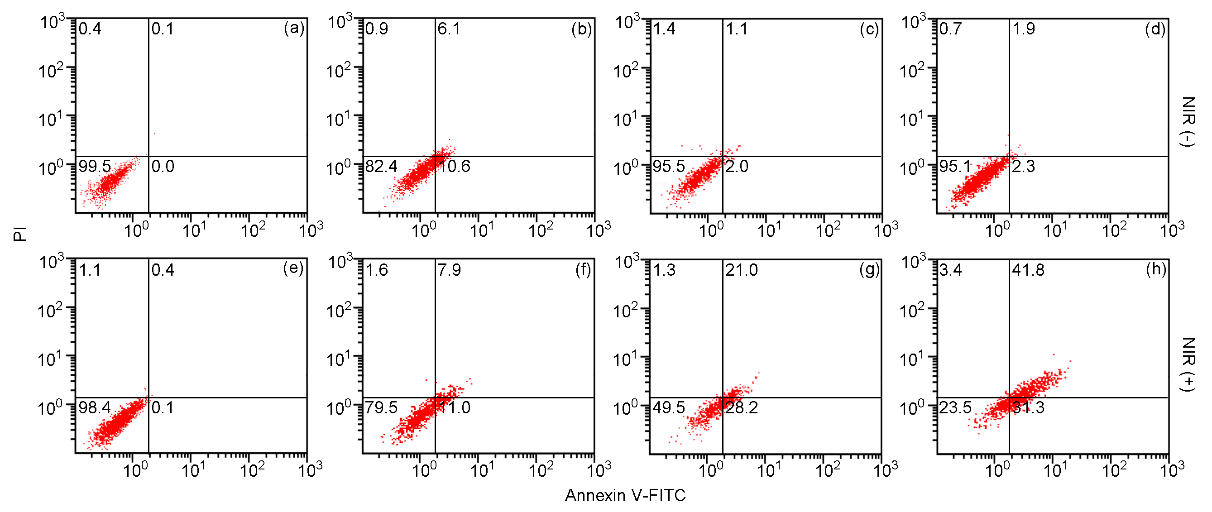
**

**Figure S18.** Flow cytometric assays of (a) and (e) MCF-7 cells, and MCF-7 cells incubated with (b) and (f) quercetin, (c) and (g) AuNPs-DNS or (d) and (h) AuNPs-DNS/Que in the (a-d) absence or (e-h) presence of 10-min NIR light irradiation.


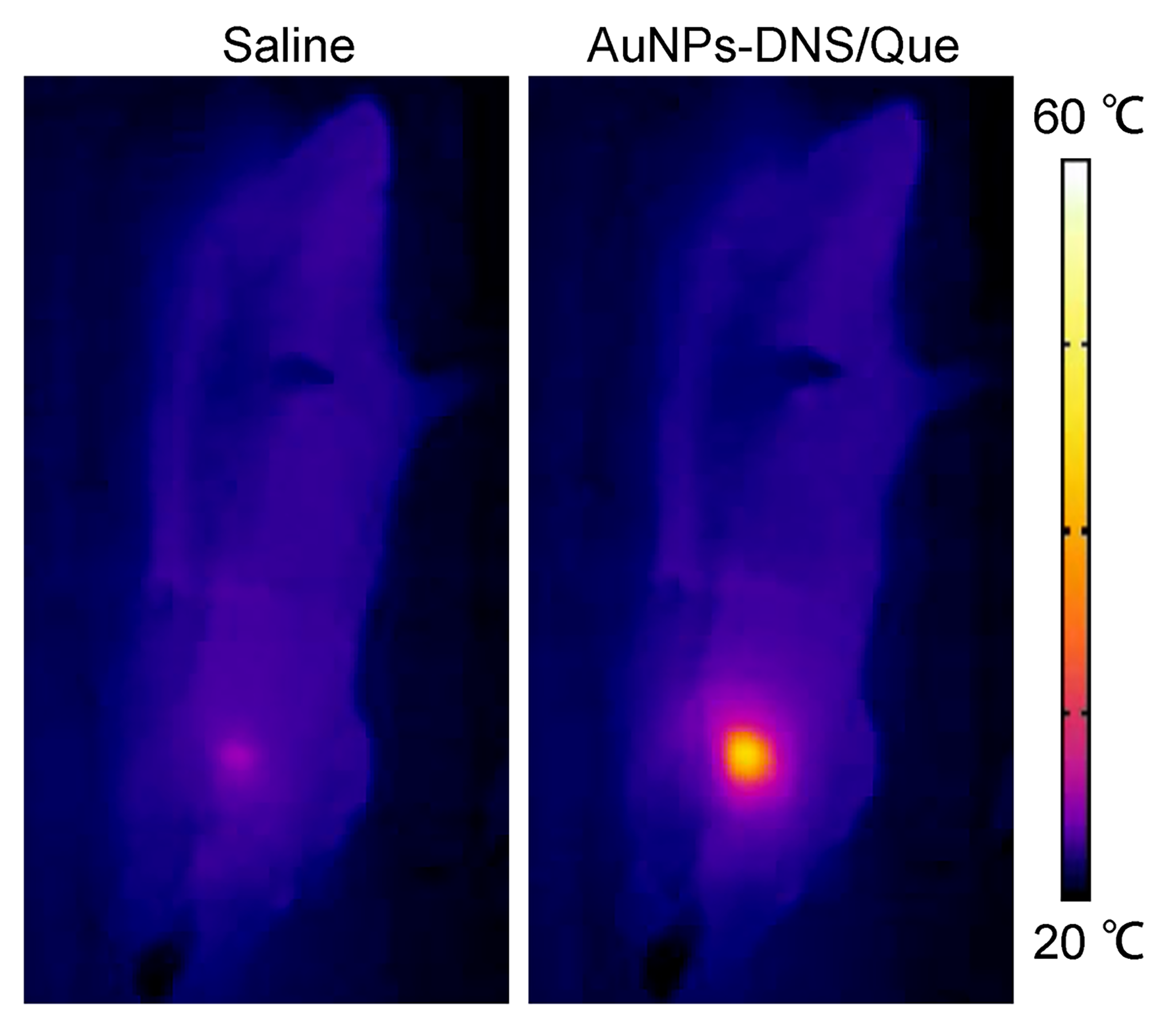


**Figure S19.** Thermographic images of mice treated with PBS or

**
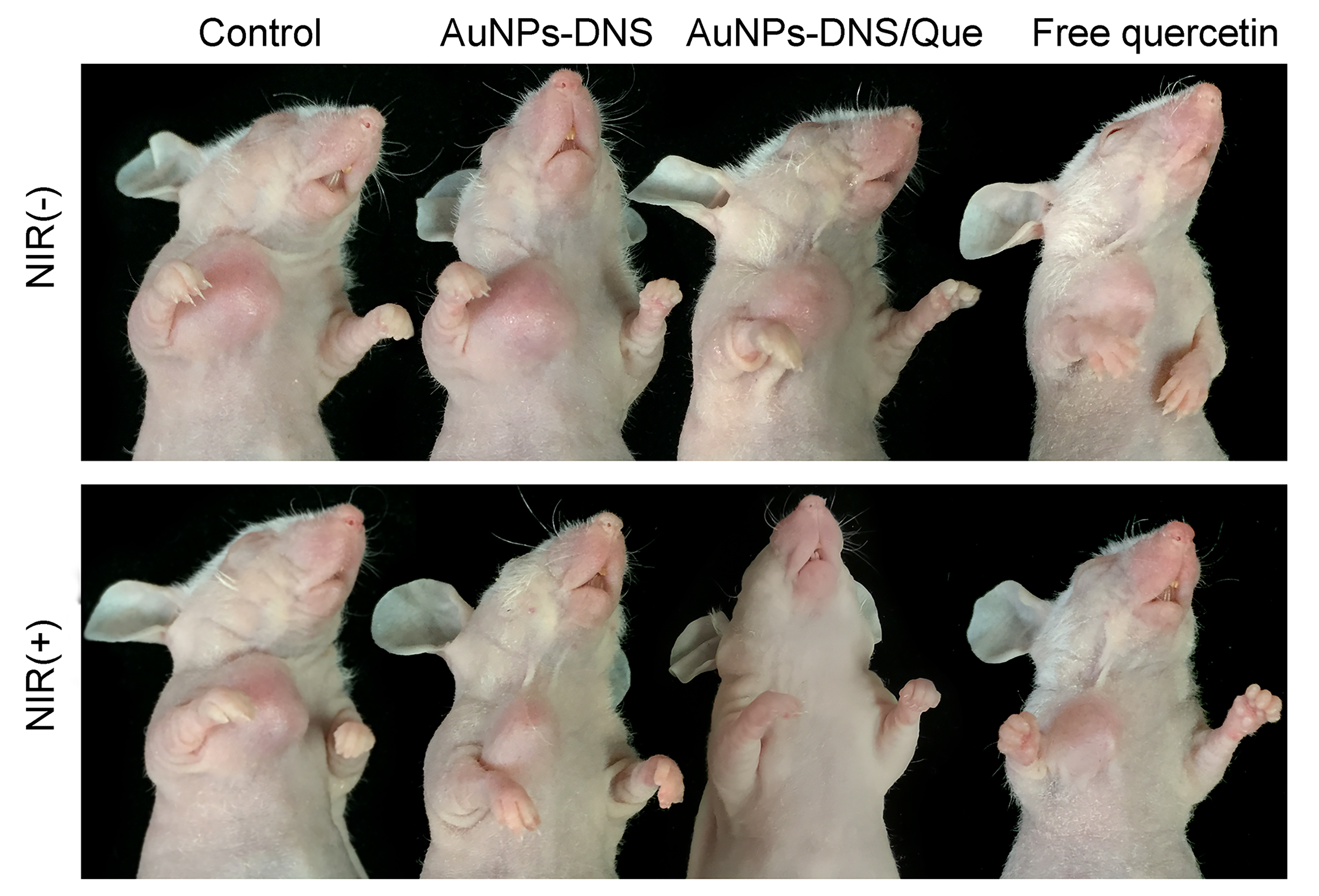
**

**Figure S20.** Representative images of mice treated with saline, AuNPs-DNS, AuNPs-DNS/Que or free quercetin with or without 800-nm light irradiation at Day 14.

**
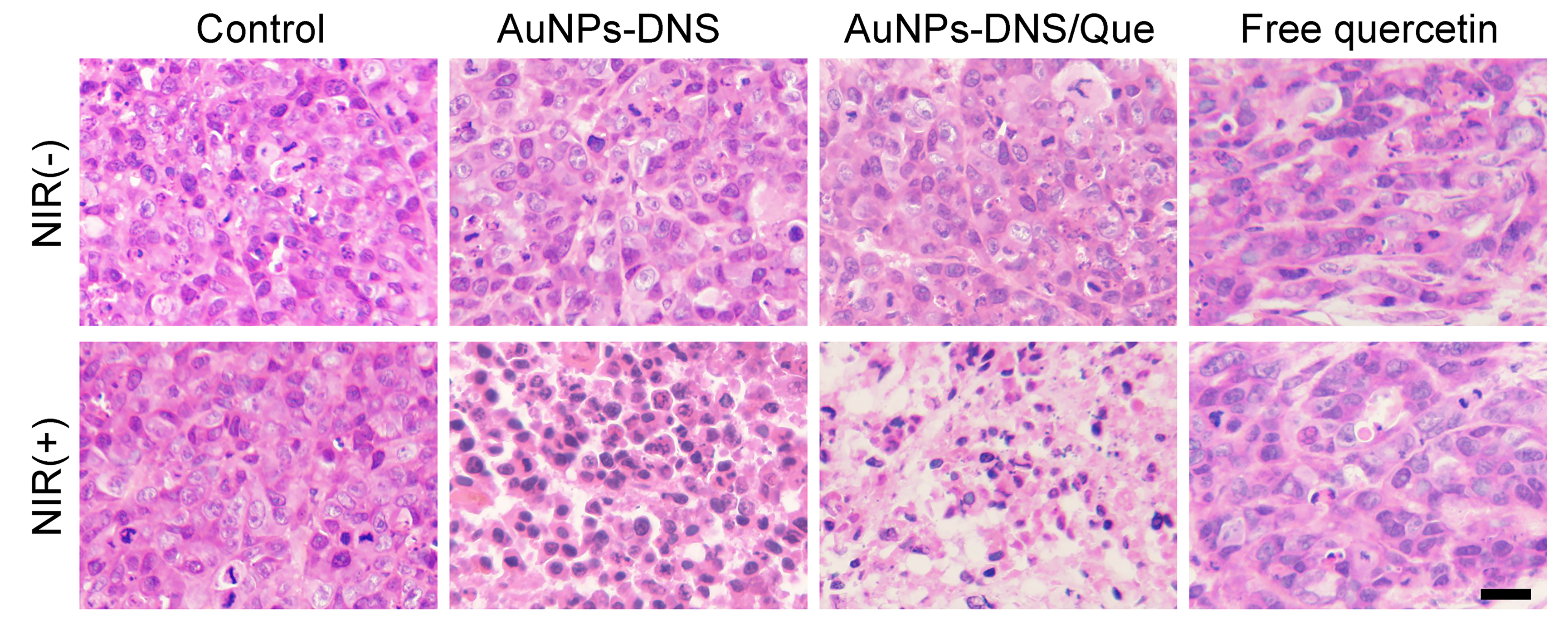
**

**Figure S21.** Histological observations of tumor tissues after treatments with saline, AuNPs-DNS, AuNPs-DNS/Que or free quercetin in the absence or presence of NIR light exposure. The scale bar indicates 100 μm.


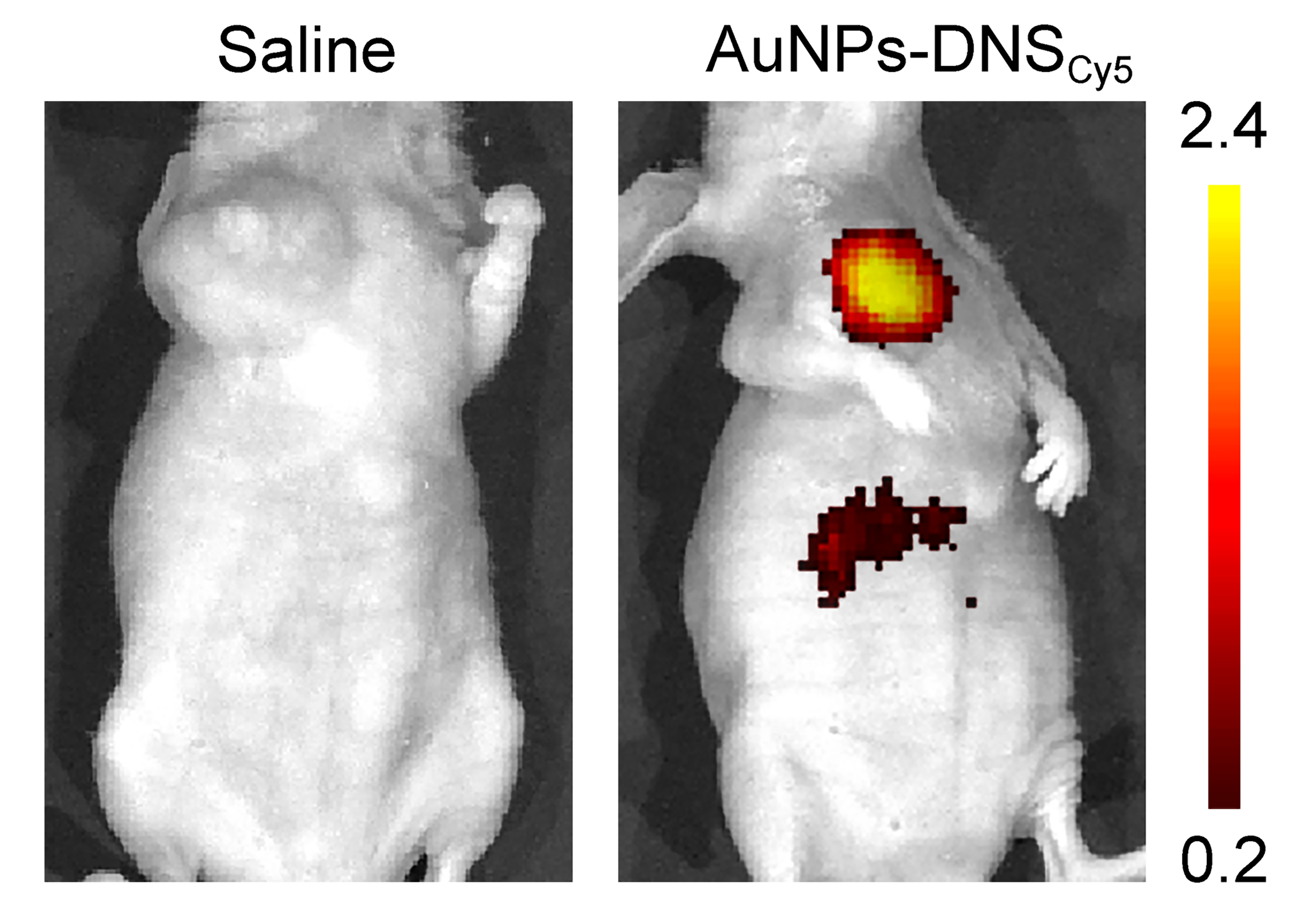


**Figure S22.** *In vivo* fluorescence imaging of mice treated with PBS or AuNPs-DNS_Cy5_.

**

**

**Figure S23.** Changes in body weight of mice treated with saline (1), AuNPs-DNS (2), AuNPs-DNS/Que (3) or free quercetin (4) with or without 800-nm light irradiation. The data error bars indicate means ± SD (*n* = 5).
